# Clinical Manifestations Associated With RT‐PCR–Confirmed Zika Virus Infection Among Adults With Acute Rash in the Brazilian Amazon: A Cross‐Sectional Study

**DOI:** 10.1002/jmv.71109

**Published:** 2026-09-07

**Authors:** Djane Clarys Baía‐da‐Silva, Yanka Karolinna Batista Rodrigues, Karolina Jeaneth Solórzano‐Chavarría, Luís Felipe Alho da Silva, Alexandre Vilhena Silva‐Neto, Luiz Henrique Gonçalves Maciel, Maria Paula Gomes Mourão, Marcia da Costa Castilho, Camila Botto‐Menezes

**Affiliations:** ^1^ Instituto Leônidas & Maria Deane (ILMD/Fiocruz Amazônia) Manaus Brazil; ^2^ Programa de Pós‐graduação em Saúde e Inovação Universidade Nilton Lins Manaus Brazil; ^3^ Instituto de Ciências Biológicas Universidade Federal de Minas Gerais Belo Horizonte Brazil; ^4^ Universidade do Estado do Amazonas Manaus Amazonas Brazil; ^5^ Departamento de Saúde Coletiva Universidade Federal do Amazonas (UFAM) Manaus Brazil; ^6^ Heitor Vieira Dourado Tropical Medicine Foundation (FMT‐HVD) Manaus Brazil

**Keywords:** arboviruses, clinical diagnosis, exanthema, predictive value, rash, Zika virus

## Abstract

Zika virus (ZIKV) infection remains a diagnostic challenge in regions where multiple arboviruses co‐circulate, particularly when laboratory testing is limited. Identifying distinguishing clinical features is therefore relevant for early case recognition. We conducted a cross‐sectional study including 1124 adults presenting with acute exanthema at a reference center in the Brazilian Amazon (2017–2022). Clinical features were assessed at the initial consultation. Diagnostic performance measures, including sensitivity, specificity, positive and negative predictive values, and accuracy, were calculated using RT‐qPCR as the reference standard. A multivariable logistic regression model including clinical, demographic, and hematological variables was constructed, and its diagnostic performance was assessed using ROC curve analysis. Among participants, 319 (28.4%) had RT‐qPCR–confirmed ZIKV infection. Pruritus (94%) and conjunctivitis (73%) were more frequent among individuals with confirmed infection. Lymphadenopathy showed the highest positive predictive value (90.9%), while pruritus had the highest negative predictive value (95.9%). Conjunctivitis demonstrated the highest diagnostic accuracy (73.3%). The multivariable model showed high apparent discrimination in the analyzed dataset (AUC = 0.94; 95% CI: 0.90–0.98), although this estimate should be interpreted cautiously because the model was not validated. Pruritus and conjunctivitis were the manifestations most consistently associated with RT‐qPCR–confirmed ZIKV infection and may support syndromic clinical suspicion in settings with limited laboratory capacity, although they do not replace laboratory confirmation. Other clinical features, including edema and arthralgia, were common but did not retain independent associations after adjustment.

## 1 Background

The Zika virus (ZIKV), which causes Zika disease, has emerged as an arbovirus of clinical and epidemiological relevance, mainly due to its association with serious neurological outcomes [1, 2, 3, 4, 5]. Starting with the first major outbreak documented on Yap Island in 2007, ZIKV spread rapidly, culminating in an epidemic of international proportions in the American region, particularly in Brazil, with repercussions, especially in the Northeast region [6, 7, 8, 9]. Although case reporting has declined globally after the epidemic peak, ZIKV continues to exhibit endemic transmission in many areas, including the disease's epicenter, with localized outbreaks, such as those reported in India and Thailand [10]. In the American region, the situation remains under surveillance, as the virus can cause recurring localized outbreaks that present an ongoing diagnostic challenge [11]. In 2024, 2117 cases were confirmed in the area up to Epidemiological Week 23, with Brazil accounting for the vast majority (97.6%) of reported cases, totaling 43,234 notifications, of which 4.8% were laboratory‐confirmed [12].

Its main clinical concern is fetal complications in pregnant women, including microcephaly and other severe brain abnormalities in fetuses of infected mothers, and the development of Guillain‐Barré syndrome in adults, an autoimmune condition that can lead to paralysis [12, 13, 14, 15, 16]. Despite having associated complications, ZIKV infection most often causes asymptomatic infection or self‐limiting acute exanthematous disease, lasting up to 7 days [17, 18, 19, 20, 21]. The most common signs and symptoms include low‐grade fever, Pruritus, Arthralgia, and conjunctivitis [22, 23, 24]. Due to the nonspecific symptoms, detection is difficult, resulting in underreporting and, consequently, limiting the availability of up‐to‐date epidemiological data on ZIKV globally [25, 26, 27]. Routine surveillance and reporting systems are insufficient in many countries, and available data often rely on clinical reports, imported cases, and research studies.

Given the regions in Brazil where arboviruses co‐circulate, the often‐nonspecific symptoms, and the limited resources for laboratory testing, the importance of carefully assessing patients’ clinical signs becomes even clearer. Therapeutic decisions rely on accurate identification of symptomatic patterns to guide the management of diseases with potentially severe outcomes, such as dengue. In this regard, recognizing clinical patterns may support early suspicion of ZIKV infection among adults with acute rash, especially where molecular testing is limited. The goal of this study was to evaluate the positive and negative predictive values (NPVs), as well as the accuracy, of clinical signs in patients with ZIKV exanthema treated at a health unit in Manaus from 2017 to 2022. This study was not designed to distinguish ZIKV infections from other arboviruses, such as dengue or chikungunya. The main purpose was to evaluate the diagnostic performance of clinical signs in identifying ZIKV infection confirmed by reverse transcription‐polymerase chain reaction (RT‐PCR) among patients presenting with rash.

## 2 Methods

### 2.1 Study Design

We conducted a cross‐sectional study at the Fundação de Medicina Tropical Doutor Heitor Vieira Dourado (FMT‐HVD) in Manaus, Amazonas, Brazil, including adults presenting with acute rash who were tested for ZIKV infection by RT‐qPCR between March 2017 and December 2022. No participants were enrolled in 2020 due to the COVID‐19 pandemic that started in Manaus.

### 2.2 Participants

Eligible participants were adults (≥ 18 years) presenting with rash for less than 7 days, who were not pregnant and provided written informed consent. Pregnant women were excluded because they were enrolled in a separate prospective study.

### 2.3 Data Collection and Laboratory Procedures

Patients presenting rash symptoms spontaneously sought care at FMT‐HVD and were invited to participate in ZIKV screening studies conducted by the Virology Department. After written informed consent was obtained, participants were evaluated by the study team for clinical assessment and medical examination. Blood and urine samples were collected for ZIKV RT‐qPCR testing, and blood samples were obtained for automated complete blood count analysis performed at the FMT‐HVD clinical laboratory. In 2017, testing followed the Lanciotti protocol; in 2018 and 2022, the Molecular ZDC kit (Bio‐Manguinhos/Fiocruz) was used [28]. A pregnancy test was performed for women of childbearing potential.

Clinical manifestations were recorded using structured forms routinely used at the time of participant enrollment. However, the availability of data for each symptom varied both between different variables and over time, reflecting differences in form completion and/or information gathering during the study period. For this reason, the analyses of each manifestation were performed considering only the number of participants for whom information was available on that specific variable.

The clinical signs and symptoms assessed were pruritus, fever (axillary temperature ≥ 37.8°C), arthralgia, conjunctivitis, edema, headache, myalgia, nausea, vomiting, diarrhea, lymphadenopathy, retro‐orbital pain, and bleeding. Because symptom availability varied across variables, analyses for manifestations with substantial missingness or very small denominators were considered exploratory.

The reference values for hematological parameters were as follows: leukocytes: 4000–10,000/mm^3^; lymphocytes: 25.0%–40.0%; platelets: 150,000–450,000/mm^3^; and hematocrit: 36.0%–47.0%. Leukopenia was defined as ≤ 3999 leukocytes/mm^3^; lymphopenia as ≤ 24.9% lymphocytes; thrombocytopenia as ≤ 149,999 platelets/mm^3^; and hemoconcentration as hematocrit ≥ 47.1%.

All clinical and laboratory data were recorded using a database managed with the Research Electronic Data Capture (REDCap) tools, hosted by FMT‐HVD [29, 30].

### 2.4 Definitions

Rash (exanthema) was defined as a generalized maculopapular skin eruption observed during clinical examination. Conjunctivitis was defined as nonpurulent conjunctival hyperemia observed during clinical examinations. Arthralgia was defined as patient‐reported joint pain affecting one or more joints. Edema was defined as visible soft‐tissue swelling identified during clinical examination.

### 2.5 Outcomes

The primary outcomes were sensitivity, specificity, positive predictive value (PPV), NPV, and diagnostic accuracy of individual signs and selected symptom combinations for RT‐qPCR‐confirmed ZIKV infection. Participants considered “Zika negative” were those who, at the time of evaluation, tested negative for ZIKV using RT‐qPCR. However, it is important to note that this classification does not rule out infection by other arboviruses (e.g., dengue or chikungunya), as systematic laboratory testing for other viruses in this family was not performed.

### 2.6 Statistical Analysis

Descriptive analyses were performed for sociodemographic, clinical, and laboratory characteristics, stratified by RT‐qPCR‐confirmed ZIKV infection status (positive vs. negative). Categorical variables were presented as absolute and relative frequencies, while continuous variables were described using medians and interquartile ranges. The number of nonmissing observations was reported for each variable. Group comparisons were conducted using Fisher's exact test or Pearson's chi‐square test with simulated *p*‐values for categorical variables, and the Wilcoxon rank‐sum test for continuous variables.

The diagnostic performance of individual clinical manifestations and selected symptom combinations was evaluated using standard accuracy measures, considering RT‐qPCR as the reference standard. For each variable, 2 × 2 contingency tables were constructed, and participants were classified as true positives (TP), false positives (FP), false negatives (FN), or true negatives (TN). Sensitivity, specificity, positive predictive value (PPV), negative predictive value (NPV), and overall accuracy were calculated. Sensitivity was defined as TP/(TP + FN), specificity as TN/(TN + FP), PPV as TP/(TP + FP), NPV as TN/(TN + FN), and accuracy as (TP + TN)/(TP + FP + FN + TN). Ninety‐five percent confidence intervals (95% CI) for sensitivity, specificity, PPV, and NPV were estimated using the epiR package (epi. tests function), while confidence intervals for accuracy were calculated using the prop. test function.

When any cell in the contingency table contained a zero value, the Haldane–Anscombe correction was applied by adding 0.5 to all cells. Analyses were restricted to participants with complete data for each evaluated variable.

For exploratory purposes, logistic regression analyses were performed to investigate possible associations between certain clinical manifestations and ZIKV infection confirmed by RT‐PCR. In constructing multivariate models, variables were selected based on both data completeness and clinical relevance. The discriminatory capacity of the multivariate model was evaluated using receiver operating characteristic (ROC) positive predictive value curve analysis. Predicted probabilities were obtained from the adjusted logistic regression model, and the area under the curve (AUC) and its 95% confidence interval were estimated using the DeLong method implemented in the pROC package. The ROC curve was constructed using the multivariate model that included the following variables: sex, age, pruritus, fever, arthralgia, conjunctivitis, edema, headache, myalgia, lymphopenia, leukopenia, thrombocytopenia, and hemoconcentration. It should be noted that the analyses were limited to complete cases, which may have generated selection bias and, consequently, influenced the model's performance estimates.

Composite variables formed from the combination of symptoms (such as pairs or triads) were not incorporated into multivariate models. Including this type of variable tends to generate redundancies and accentuate collinearities, since it adds information already present in the individual predictors. To preserve both the stability and interpretative clarity of the model, it was decided to retain only the individual clinical variables in the adjusted analyses.

Statistical analyses were performed using RStudio (version 4.4; R Foundation for Statistical Computing). Data management was conducted using the dplyr package, and summary and comparison tables were generated with the gtsummary package.

### 2.7 Ethical Aspects

Ethical approval was obtained from the Research Ethics Committee of FMT‐HVD (CAAE: 80961517.6.000.0005). All participants provided written informed consent.

## 3 Results

The study included 1124 participants with acute exanthema, of whom 319 (28.4%) had ZIKV infection confirmed by RT‐qPCR. Confirmed cases of ZIKV infection were predominantly concentrated in the early years of the study period. There were positive records in 2017, 2018, and 2019. No RT‐qPCR‐confirmed cases were identified among participants included in 2021 and 2022 (Supporting Information: Table S1).

Table 1 summarizes the demographic, clinical, and laboratory characteristics of participants according to ZIKV infection status. The proportion of women was significantly higher among ZIKV‐positive individuals compared with those testing negative (71% vs. 51%, *p* < 0.001). Differences were also observed in the distribution of race/color categories (*p* < 0.001), although brown (mixed‐race) individuals predominated in both groups. The median age was similar across groups, approximately 36 years.

**Table 1:** Demographic, clinical, and hematological characteristics of adults with acute exanthema according to RT‐qPCR–confirmed ZIKV infection status, and crude and adjusted associations with infection.

	Zika positive (*n* = 319) *n* (%) or Median (Q1‐Q3)^a^	Zika negative (*n* = 805) *n* (%) or Median (Q1‐Q3)^a^	Total (*n* = 1,124) *n* (%) or Median (Q1‐Q3)^a^	Univariate^b^			Multivariable		
				OR	95% CI	*p*‐value	OR	95% CI	*p*‐value
Age (years)^c^	36 (27–46)	37 (27–48)	36 (27–48)	0.99	0.98, 1.00	0.122	0.97	0.93, 1.01	0.124
Gender									
Male	93/319 (29%)	395/805 (49%)	488/1124 (43%)	—	—	—	—	—	—
Female	226/319 (71%)	410/805 (51%)	636/1,124 (57%)	2.34	1.78, 3.10	< 0.001	1.1	0.33, 4.08	0.877
Race/color									
White	41/256 (16%)	96/496 (19%)	137/752 (18%)	—	—	—	—	—	—
Black	9/256 (3.5%)	38/496 (7.7%)	47/752 (6.3%)	0.55	0.23, 1.21	0.155	—	—	—
Brown/pardo (mixed race)	177/256 (69%)	335/496 (68%)	512/752 (68%)	1.24	0.83, 1.88	0.307	—	—	—
Indigenous	7/256 (2.7%)	24/496 (4.8%)	31/752 (4.1%)	0.68	0.26, 1.64	0.415	—	—	—
Asian	0/256 (0%)	3/496 (0.6%)	3/752 (0.4%)	—	—	—	—	—	—
Other	9/256 (3.5%)	0/496 (0%)	9/752 (1.2%)	0		0.991	—	—	—
Does not know	13/256 (5.1%)	0/496 (0%)	13/752 (1.7%)	—	—	—	—	—	—
Education (years)	11.0 (11.0‐15.0)	NA (NA‐NA)	11.0 (11.0‐15.0)	—	—	—	—	—	—
Postpartum	1/31 (3.2%)	1/14 (7.1%)	2/45 (4.4%)	0.43	0.02, 11.5	0.565	—	—	—
Breastfeeding	7/132 (5.3%)	9/142 (6.3%)	16/274 (5.8%)	0.83	0.29, 2.29	0.715	—	—	—
Days of symptoms^d^	3.00 (2.00–4.00)	4.00 (3.00–6.00)	4.00 (2.00–6.00)	0.88	0.85, 0.95	< 0.001	—	—	—
Temperature (°C)	36.80 (36.40–37.30)	36.70 (36.30–37.00)	36.70 (36.40–37.00)	1.5	1.24, 1.82	< 0.001	—	—	—
Signs or symptoms									
Pruritus	300/319 (94%)	365/802 (46%)	665/1121 (59%)	18.9	12.0, 31.7	< 0.001	10.7	2.69, 54.3	0.001
Fever	265/318 (83%)	749/802 (93%)	1014/1,120 (91%)	0.35	0.24, 0.53	< 0.001	0.3	0.07, 1.27	0.098
Arthralgia	258/319 (81%)	513/802 (64%)	771/1121 (69%)	2.38	1.75, 3.28	< 0.001	0.76	0.22, 2.82	0.674
Conjunctivitis	231/317 (73%)	212/800 (27%)	443/1117 (40%)	7.45	5.58, 10.0	< 0.001	4.88	1.73, 14.8	0.004
Edema	216/316 (68%)	235/798 (29%)	451/1114 (40%)	5.17	3.91, 6.88	< 0.001	1	0.32, 2.99	0.993
Headache	24/33 (73%)	476/505 (94%)	500/538 (93%)	0.16	0.07, 0.40	< 0.001	0.7	0.19, 2.72	0.592
Myalgia	23/33 (70%)	438/506 (87%)	461/539 (86%)	0.36	0.17, 0.82	0.01	0.29	0.09, 0.93	0.037
Nausea	15/33 (45%)	6/9 (67%)	21/42 (50%)	0.42	0.08, 1.86	0.267	—	—	—
Vomiting	6/33 (18%)	125/506 (25%)	131/539 (24%)	0.68	0.25, 1.57	0.4	—	—	—
Diarrhea	7/32 (22%)	128/503 (25%)	135/535 (25%)	0.82	0.32, 1.85	0.652	—	—	—
Lymphadenopathy	10/31 (32%)	1/6 (17%)	11/37 (30%)	2.38	0.32, 48.8	0.455	—	—	—
Retro‐orbital pain	3/5 (60%)	224/500 (45%)	227/505 (45%)	1.85	0.30, 14.1	0.503	—	—	—
Bleeding	1/1 (100%)	25/494 (5.1%)	26/495 (5.3%)	—	—	—	—	—	—
Laboratory findings									
Leukocytes^e^	4570 (3720–5980)	6099 (4171–8390)	5458 (3910–7800)	1	1.00, 1.00	< 0.001	—	—	—
Leukopenia	81/245 (33%)	135/597 (23%)	216/842 (26%)	1.69	1.22, 2.34	0.002	2.39	0.79, 7.38	0.124
Lymphocytes^f^	33 (26–40)	26 (16–35)	28 (19–37)	1.04	1.03, 1.05	< 0.001	—	—	—
Lymphopenia	58/245 (24%)	284/597 (48%)	342/842 (41%)	0.34	0.24, 0.48	< 0.001	0.83	0.28, 2.38	0.734
Platelets^g^	228,000 (194,000, 276,000)	195,900 (134,900, 248,600)	208,050 (154,400–258,700)	1	1.00, 1.00	< 0.001	—	—	—
Thrombocytopenia	20/245 (8.2%)	174/597 (29%)	194/842 (23%)	0.22	0.13, 0.34	< 0.001	0.11	0.02, 0.47	0.009
Hematocrit^h^	40.1 (37.8–42.8)	42.1 (38.4–45.4)	41.3 (38.3–45.0)	0.94	0.92, 0.97	< 0.001	—	—	—
Hemoconcentration	230/245 (94%)	498/597 (83%)	728/842 (86%)	3.05	1.78, 5.57	< 0.001	4.15	0.70, 37.7	0.15

*Note:* Denominators vary across variables because symptom‐level data was unavailable for all participants. Nausea had particularly high missingness, especially in the ZIKV‐negative group. Bleeding data were available for only one participant in the ZIKV‐positive group; therefore, this variable should be considered descriptive only and not suitable for inferential comparison. Percentages were calculated using the number of participants with available data for each variable.

^a^ Median (Q1–Q3); *n* (%).

^b^ Wilcoxon rank sum test or Chi‐squared tests or Fisher's Exact Test.

^c^ Maximum value: 88 years.

^d^ Minimum value: 1 day; maximum value: 14 days.

^e^ Minimum value: 1200 leukocytes/mm^3^; maximum value: 27,240 leukocytes/mm^3^.

^f^ Minimum value: 0% lymphocytes; maximum value: 70% lymphocytes.

^g^ Minimum value: platelets/mm^3^; maximum value: 721,000 platelets/mm^3^.

^h^ Minimum value: 22.2%; maximum value: 58.3%.

Given the limited number of observations for some clinical variables, such as bleeding, nausea, retro‐orbital pain, and lymphadenopathy, it is important that analyses involving these outcomes be viewed as exploratory in nature. Small samples, after all, can result in unstable estimates, limiting the accuracy of the results related to these manifestations.

Several clinical manifestations were more frequent among participants with confirmed ZIKV infection. Pruritus (94% vs. 46%, *p* < 0.001), conjunctivitis (73% vs. 27%, *p* < 0.001), edema (68% vs. 29%, *p* < 0.001), and arthralgia (81% vs. 64%, *p* < 0.001) showed the largest absolute differences between groups. In contrast, fever was more frequently observed among ZIKV‐negative participants (93% vs. 83%, *p* < 0.001).

Among pairwise combinations, pruritus plus conjunctivitis (68% vs. 20%, *p* < 0.001), pruritus plus arthralgia (77% vs. 34%, *p* < 0.001), and conjunctivitis plus edema (53% vs. 15%, *p* < 0.001) were substantially more frequent among ZIKV‐positive individuals. Among triads, the combinations of pruritus, conjunctivitis, and arthralgia (58% vs. 17%, *p* < 0.001), pruritus, conjunctivitis, and edema (50% vs. 13%, *p* < 0.001), and pruritus, arthralgia, and edema (58% vs. 20%, *p* < 0.001) were also more common in the ZIKV‐positive group (Supporting Information: Table S2).

Compared to ZIKV‐negative participants, those infected presented lower median leukocyte counts (4570/mm^3^ vs. 6099/mm^3^, *p* < 0.001), a higher occurrence of leukopenia (33% vs. 23%, *p* = 0.002), a lower proportion of lymphopenia (24% vs. 48%, *p* < 0.001), and a higher platelet count (228,000/mm^3^ vs. 195,900/mm^3^, *p* < 0.001), in addition to presenting less thrombocytopenia (8.2% vs. 29%, *p* < 0.001) and hemoconcentration (6.1% vs. 17%, *p* < 0.001).

The diagnostic performance of individual clinical manifestations and selected combinations is presented in Table 2 and Supporting Information: Table S3.

**Table 2:** Diagnostic performance of selected clinical manifestations for identifying RT‐qPCR–confirmed ZIKV infection among adults presenting with acute exanthema.

Signs and symptoms	TP^a^	FP^b^	FN^c^	TN^d^	Sensitivity % (95% CI)^e^	Specificity % (95% CI)^e^	PPV^f^% (95% CI)^e^	NPV^g^% (95% CI)^e^	Accuracy % (95% CI)^e^
Fever	265	749	53	53	83.3 (78.8–87.3)	6.6 (5–8.6)	26.1 (23.5–29)	50 (40.1–59.9)	28.4 (25.8–31.1)
Pruritus	300	365	19	437	94 (90.9–96.4)	54.5 (51–58)	45.1 (41.3–49)	95.8 (93.6–97.5)	65.7 (62.9–68.5)
Conjunctivitis	231	212	86	588	72.9 (67.6–77.7)	73.5 (70.3–76.5)	52.1 (47.4–56.9)	87.2 (84.5–89.7)	73.3 (70.6–75.9)
Arthralgia	258	513	61	289	80.9 (76.1–85)	36 (32.7–39.5)	33.5 (30.1–36.9)	82.6 (78.2–86.4)	48.8 (45.8–51.8)
Edema	216	235	100	563	68.4 (62.9–73.4)	70.6 (67.3–73.7)	47.9 (43.2–52.6)	84.9 (82–87.6)	69.9 (67.1–72.6)
Headache	24	476	9	29	72.7 (54.5–86.7)	5.7 (3.9–8.1)	4.8 (3.1–7.1)	76.3 (59.8–88.6)	9.9 (7.5–12.8)
Myalgia	23	438	10	68	69.7 (51.3–84.4)	13.4 (10.6–16.7)	5 (3.2–7.4)	87.2 (77.7–93.7)	16.9 (13.9–20.4)
Lymphadenopathy	10	1	21	5	32.3 (16.7–51.4)	83.3 (35.9–99.6)	90.9 (58.7–99.8)	19.2 (6.6–39.4)	40.5 (25.2–57.8)
Diarrhea	7	128	25	375	21.9 (9.3–40)	74.6 (70.5–78.3)	5.2 (2.1–10.4)	93.8 (90.9–95.9)	71.4 (67.3–75.2)
Nausea	15	6	18	3	45.5 (28.1–63.6)	33.3 (7.5–70.1)	71.4 (47.8–88.7)	14.3 (3–36.3)	42.9 (28.1–58.9)
Vomiting	6	125	27	381	18.2 (7–35.5)	75.3 (71.3–79)	4.6 (1.7–9.7)	93.4 (90.5–95.6)	71.8 (67.8–75.5)

*Note:* Diagnostic estimates for less frequent manifestations, particularly lymphadenopathy, should be interpreted cautiously because of the small number of observations.

^a^ True positive.

^b^ False positive.

^c^ False negative.

^d^ True negative.

^e^ 95% confidence interval.

^f^ Positive predictive value.

^g^ Negative predictive value.

Among isolated clinical manifestations, pruritus showed the highest sensitivity (94.0%) but only moderate specificity (54.5%), indicating that it was useful for identifying potential cases but had limited discriminatory value when considered alone. Conjunctivitis showed a more balanced performance, with sensitivity of 72.9% and specificity of 73.5%, whereas edema had intermediate diagnostic utility (sensitivity 68.4%; specificity 70.6%). Arthralgia was frequent and sensitive (80.9%) but poorly specific (36.0%), and fever had very low specificity (6.6%), indicating minimal usefulness for discriminating ZIKV infection within this exanthematic population.

Symptom combinations generally yielded higher specificity compared with individual manifestations, with a corresponding reduction in sensitivity. The combination of conjunctivitis and edema showed sensitivity of 53.5%, specificity of 85.0%, and accuracy of 76.1%. Similarly, the triad of pruritus, conjunctivitis, and edema showed sensitivity of 50.3%, specificity of 87.1%, and accuracy of 76.7%, while other triads demonstrated slightly higher sensitivity but lower specificity.

In the univariate analysis, several clinical and laboratory variables were associated with RT‐PCR‐confirmed ZIKV infection, including pruritus, fever, arthralgia, conjunctivitis, edema, headache, myalgia, hematological changes, and symptom duration (Table 1). Among the clinical variables, a shorter symptom duration was associated with a ZIKV diagnosis (OR = 0.88; 95% CI: 0.85–0.95; *p* < 0.001), while higher body temperatures also showed a positive association with the outcome (OR = 1.50; 95% CI: 1.24–1.82; *p* < 0.001). Female sex was associated with a higher probability of infection by the virus (OR = 2.34; 95% CI: 1.78–3.10; *p* < 0.001), unlike age, which did not show a statistically significant association (OR = 0.99; 95% CI: 0.98–1.00; *p* = 0.122). No relevant associations were found for variables such as race/color, postpartum status, and breastfeeding.

In terms of signs and symptoms, pruritus stood out as the marker most strongly associated with ZIKV infection (OR = 18.9; 95% CI: 12.0–31.7; *p* < 0.001), followed by conjunctivitis (OR = 7.45; 95% CI: 5.58–10.0; *p* < 0.001), edema (OR = 5.17; 95% CI: 3.91–6.88; *p* < 0.001) and arthralgia (OR = 2.38; 95% CI: 1.75–3.28; *p* < 0.001). Conversely, fever (OR = 0.35; 95% CI: 0.24–0.53; *p* < 0.001), headache (OR = 0.16; 95% CI: 0.07–0.40; *p* < 0.001), and myalgia (OR = 0.36; 95% CI: 0.17–0.82; *p* = 0.01) were associated with a lower probability of infection. Other symptoms, such as nausea, vomiting, diarrhea, lymphadenopathy, and retro‐orbital pain, did not show significant associations.

Regarding laboratory parameters, ZIKV infection was associated with reduced leukocyte and platelet counts and decreased hematocrit. Leukopenia was positively associated with the outcome (OR = 1.69; 95% CI: 1.22–2.34; *p* = 0.002), while lymphopenia (OR = 0.34; 95% CI: 0.24–0.48; *p* < 0.001) and thrombocytopenia (OR = 0.22; 95% CI: 0.13–0.34; *p* < 0.001) corresponded to a lower chance of infection. Hemoconcentration was positively associated with the diagnosis (OR = 3.05; 95% CI: 1.78–5.57; *p* < 0.001).

**Figure 1:**
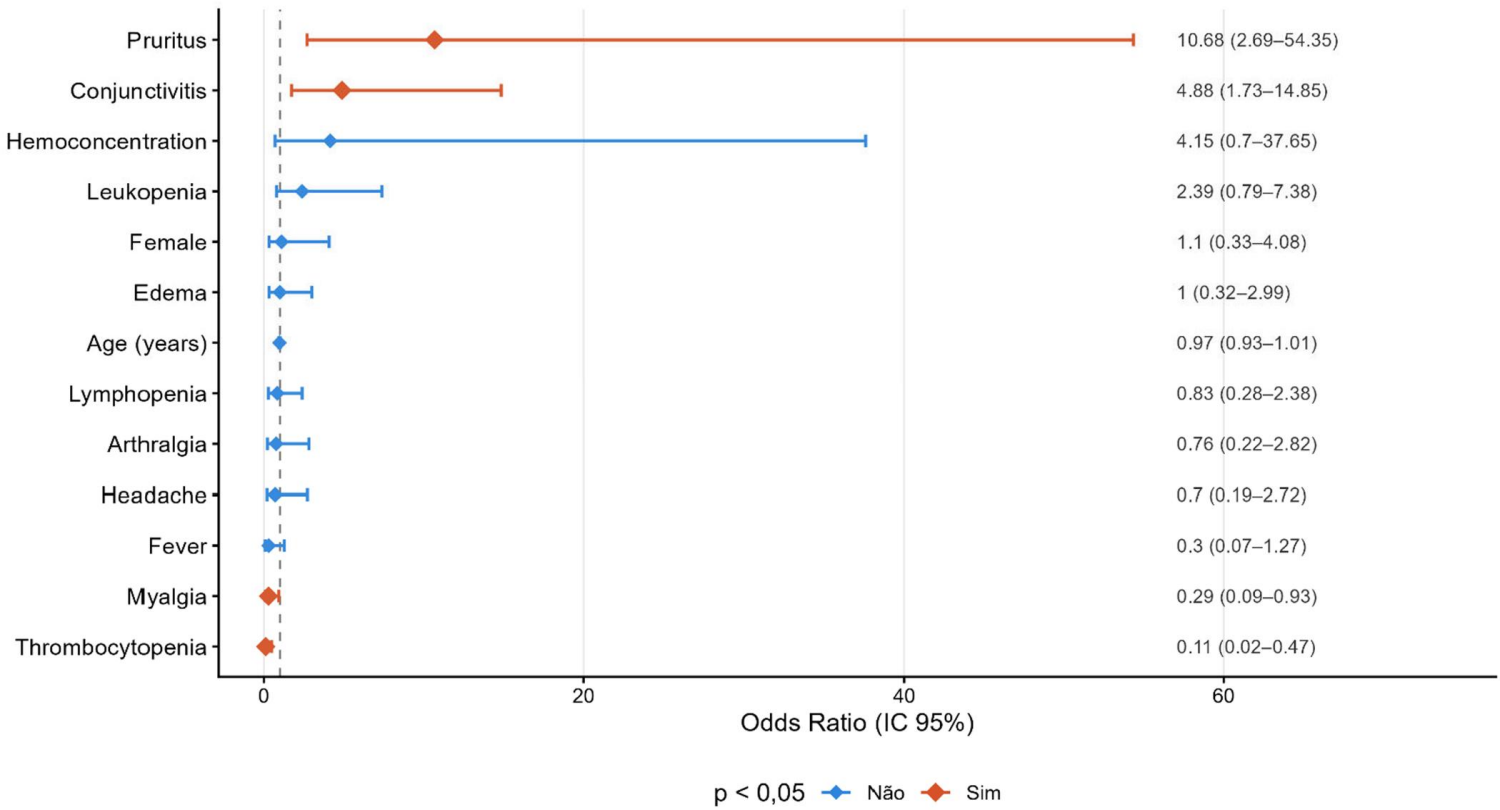
Forest plot of adjusted odds ratios (OR) for the association between selected clinical manifestations and RT‐PCR–confirmed ZIKV infection. Estimates were derived from a multivariable logistic regression model and should be interpreted as exploratory, given missing data and the use of complete‐case analysis.

After adjustment in the multivariate model, only a small set of variables remained independently associated with ZIKV infection. Pruritus had the strongest association (OR = 10.7; 95% CI: 2.69–54.3; *p* = 0.001), followed by conjunctivitis (OR = 4.88; 95% CI: 1.73–14.8; *p* = 0.004), both of which were associated with a higher risk of infection. Conversely, thrombocytopenia (OR = 0.11; 95% CI: 0.02–0.47; *p* = 0.009) and myalgia (OR = 0.29; 95% CI: 0.09–0.93; *p* = 0.037) remained associated with a lower probability of ZIKV (Table 1 and Figure 1). The other variables, such as fever, arthralgia, edema, headache, leukopenia, lymphopenia, and hemoconcentration, did not show statistical significance after adjustment.

**Figure 2:**
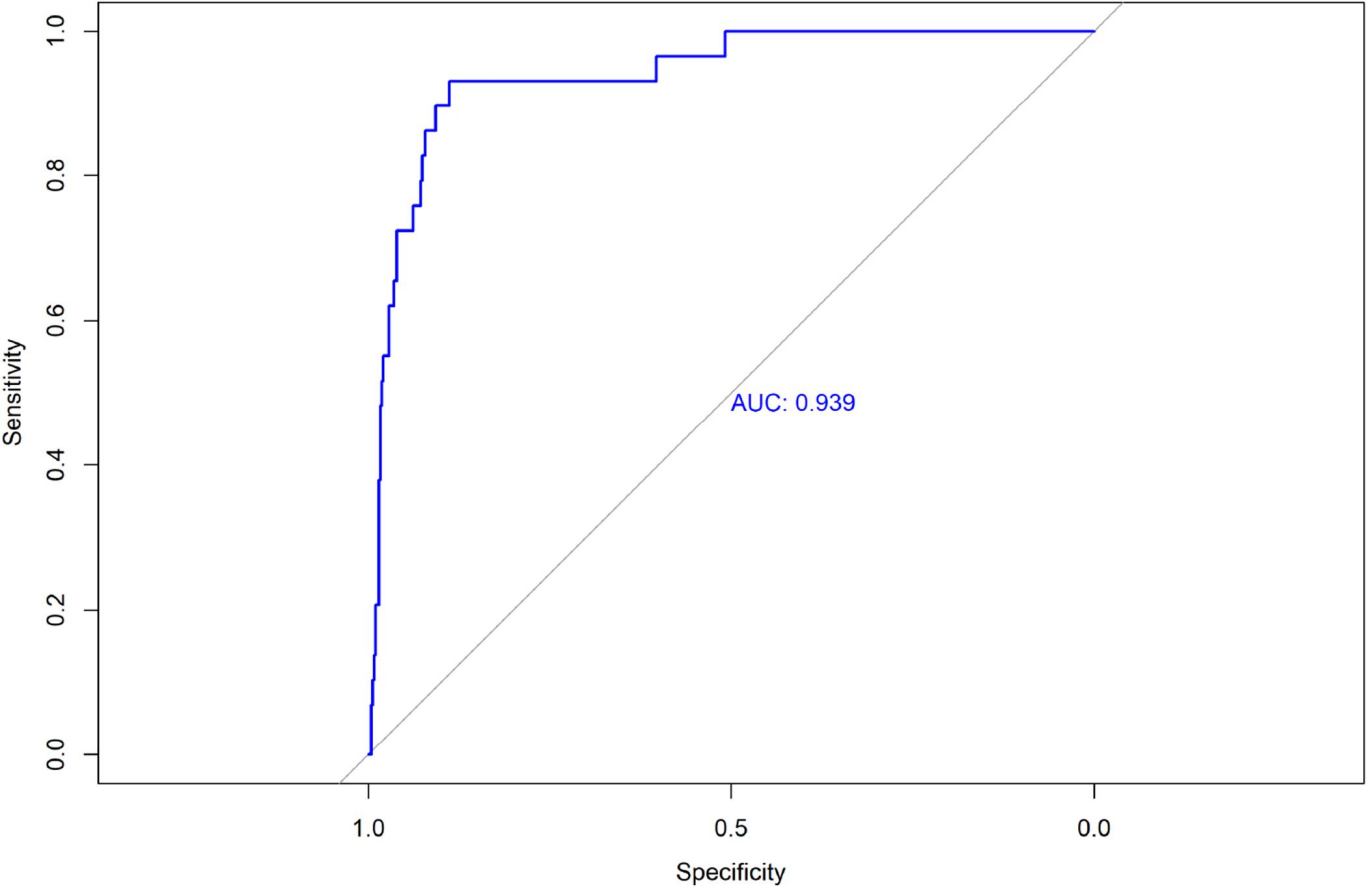
ROC curve for the multivariable logistic regression model predicting RT‐PCR–confirmed Zika virus infection. The model included sex, age, pruritus, fever, arthralgia, conjunctivitis, edema, headache, myalgia, and hematological parameters. The area under the ROC curve (AUC) was 0.94 (95% CI: 0.90–0.98). Specificity is plotted in decreasing order.

The multivariate model showed high apparent discrimination in this sample, with an area under the ROC curve of 0.94 (95% CI: 0.90–0.98), estimated using the DeLong method (Figure 2).

## 4 Discussion

In areas of the Amazon marked by intense co‐circulation of ZIKV, DENV, and CHIKV, and where access to RT‐qPCR is limited, identifying the clinical pattern associated with ZIKV infection in adults with skin rash is a fundamental resource for guiding diagnostic suspicion and rationalizing requests for laboratory tests. Previous studies have consistently shown a significant overlap between the clinical manifestations of the main arboviruses, although certain symptoms are more frequent in ZIKV cases. In case series of ZIKV infection confirmed by RT‐PCR in adults, skin rash is frequently the most common symptom, almost universal, followed by arthralgia, myalgia, usually low‐grade or absent fever, conjunctivitis, and headache, in descending order of frequency in the reports [22, 31, 32, 33, 34]. Despite this relatively homogeneous profile, research conducted in contexts of arboviral co‐circulation demonstrates that isolated clinical manifestations have limited discriminatory value. This is because quite similar symptomatic presentations are also observed in dengue and chikungunya infections [32, 33, 35, 36, 37]. Thus, the accuracy of purely clinical classification, especially in the context of surveillance, tends to be low, reinforcing the importance of laboratory confirmation whenever possible. In the present study, the results point to a recognizable, though not exclusive, clinical pattern among adults presenting with acute exanthema. It is important to emphasize, however, that the design of this study did not aim to directly discriminate ZIKV infection from other arboviruses, since participants classified as negative for ZIKV were not systematically investigated for other circulating arboviruses. For this reason, the identified differences should be understood from a syndromic perspective rather than as direct etiological comparisons.

A predominance of women was observed among the participants who tested positive for ZIKV, consistent with previous reports in Brazil and with broader analyses of ZIKV infection in adults [38, 39, 40, 41, 42]. This difference may reflect, at least in part, variations in healthcare‐seeking behavior, symptom perception, or case‐finding mechanisms, rather than a greater biological susceptibility [39, 40]. The lack of association with age reinforces the nonspecific nature of ZIKV infection across different adult age groups.

In this cross‐sectional study of adults treated for acute exanthema in the Brazilian Amazon, two clinical signs consistently stood out after multivariate adjustment: pruritus and conjunctivitis remained strongly associated with ZIKV infection confirmed by RT‐PCR. Although edema and arthralgia appeared more frequently in positive cases in initial analyses, these associations weakened when other factors were considered. Overall, the results reveal a recognizable clinical profile of Zika infection in this context. However, this pattern, while relevant, is not specific enough to eliminate the need for laboratory confirmation.

These findings are consistent with those reported in other studies on ZIKV infection in adults, in which manifestations such as rash, itching, conjunctivitis, and arthralgia are among the most frequently reported symptoms [22, 31, 33, 38]. Even so, it is worth remembering that the overlap of clinical signs between ZIKV and other arboviruses, such as dengue and chikungunya, remains significant, especially in endemic regions with simultaneous circulation of these viruses. Therefore, it is essential to consider the results of this study within a syndromic approach rather than an etiological one, since participants classified as negative for ZIKV did not undergo systematic screening for other arboviruses.

The higher frequencies of pruritus, conjunctivitis, edema, and arthralgia among those positive for ZIKV reinforce the relevance of these manifestations as clinical markers of confirmed infection. This pattern is consistent with previous descriptions of the disease in adults, in which rash, pruritus, arthralgia, and conjunctivitis are among the most frequently reported symptoms. It is noteworthy that pruritus and conjunctivitis remain significantly associated in the multivariate model, suggesting that these manifestations capture central elements of the clinical phenotype of ZIKV infection.

The fact that itching, conjunctivitis, edema, and arthralgia are more frequent among confirmed cases reinforces the importance of these signs as possible clinical markers of infection. On the other hand, the weakening of these associations after multivariate adjustment indicates that many of these symptoms may result from common clinical mechanisms or confounding factors, and do not necessarily represent exclusive diagnostic markers of ZIKV. Itching and conjunctivitis stand out, as they reflect central aspects of the typical clinical picture of viral infection.

Pruritus stood out for its high sensitivity and high NPV, suggesting it may be a valuable indicator for assessing clinical suspicion of ZIKV infection. Conjunctivitis showed a more balanced performance between sensitivity and specificity, which increases its potential as a discriminatory marker when present. On the other hand, fever showed very low specificity and, curiously, was even more common among those without laboratory confirmation of ZIKV, highlighting its limited usefulness in differentiating Zika from other febrile or exanthematous diseases in daily clinical practice. It is worth noting that this pattern is consistent with previous studies, which characterize Zika infection as frequently afebrile, or accompanied only by mild fever, in contrast to the typical presentations of dengue and Chikungunya [31, 42].

The association of symptoms proved capable of increasing specificity, even at the cost of lower sensitivity, a diagnostic phenomenon that was already expected. Although these combinations of signs are valuable in clinical decision‐making, they should not be considered absolute diagnostic criteria. It is also worth noting that certain manifestations, such as lymphadenopathy, showed apparently high predictive estimates, but were identified in very few cases and, therefore, should be viewed in an exploratory manner. The same caution applies to symptoms such as bleeding, nausea, and retro‐orbital pain, since the number of records available for these variables was small and unevenly distributed.

The results related to hematological findings also require careful interpretation. Although some differences were noted between the analyzed groups, several laboratory variables were impacted by issues such as missing data, adopted categorization criteria, and the small number of complete cases included in the adjusted analysis. Given this scenario, it is more appropriate to consider these findings as specific descriptions of the laboratory profile rather than as independent and reliable diagnostic indicators. The observed pattern, with a higher frequency of leukopenia and a lower incidence of thrombocytopenia and hemoconcentration in ZIKV‐positive cases, aligns with previous studies indicating milder hematological alterations associated with Zika than with dengue. However, it is important to emphasize that, in this context, the ability to establish causal relationships remains limited.

The multivariate model's performance, at first glance, appeared high (AUC = 0.94; 95% CI: 0.90–0.98). However, it is important to view this result with some caution, as the estimate may be overestimated. This is because the model was fitted and tested on the same dataset without any internal or external validation, and some predictors exhibited clinical correlations among themselves. For these reasons, it is essentially an exploratory model, useful for generating hypotheses but far from ready for direct application in clinical practice.

The evolution of cases over time proved uneven. Confirmed ZIKV infections were limited to the first years of the study, while in subsequent years, no new cases were observed. This is likely a reflection of the dynamics of viral circulation following the large outbreaks in the Americas [43, 44]. Consequently, the predictive values obtained are strongly influenced by local disease prevalence and should always be analyzed in the context of the current epidemiological context.

This study also presents some important limitations. For example, there was no prior calculation of the sample size, which may have compromised both statistical power and the stability of the model's estimates. The fact that only complete case analyses were used raises the possibility of selection bias. Furthermore, the absence of validation (internal or external) reduces the likelihood that the results can be generalized to other scenarios. It should also be noted that the entire investigation was conducted in a single reference center, which naturally restricts the applicability of the findings to other contexts. Another relevant point is that, although RT‐PCR was adopted as the gold standard, there is a risk of FNs due to the short duration of viremia. Added to this is the lack of systematic testing for other arboviruses, hindering a more precise etiological interpretation. Finally, it is important to remember that the predictive values extracted in this work depend directly on the local prevalence of the disease and may not fully apply to regions with different levels of ZIKV circulation.

## 5 Conclusion

This cross‐sectional study conducted among adults with acute skin rash in the Brazilian Amazon identified pruritus and conjunctivitis as the clinical signs most frequently associated with ZIKV infection confirmed by RT‐PCR. These results support the existence of a recognizable clinical profile but also make it clear that the significant overlap of symptoms with other arboviruses restricts the specificity of a purely clinical diagnosis. In practice, the presence of these signs can guide clinical reasoning and serve as a starting point for evaluating suspected cases, with conjunctivitis adding value to diagnostic differentiation. Even so, none of the symptoms analyzed in isolation proved sufficiently precise to replace laboratory confirmation. Thus, our findings reinforce the importance of a syndrome‐based approach in settings with limited diagnostic resources, while reiterating the fundamental role of laboratory tests and the need for rigorous clinical validation of the diagnosis.

## Author Contributions

**Djane Clarys Baía‐da‐Silva:** conceptualization, data curation, formal analysis, investigation, methodology, writing – original draft. **Yanka Karolinna Batista Rodrigues:** data curation, investigation, writing – review and editing. **Karolina Jeaneth Solórzano‐Chavarría:** writing – review and editing. **Luís Felipe Alhoda Silva:** investigation, data curation, writing – review and editing. **Alexandre Vilhena Silva‐Neto:** investigation, data curation, data analysis, writing – review and editing. **Luiz Henrique Gonçalves Maciel:** methodology, writing – review and editing. **Maria Paula Gomes Mourão:** supervision, resources, writing – review and editing. **Marcia da Costa Castilho:** supervision, resources, writing – review and editing. **Camila Botto‐Menezes:** conceptualization, methodology, supervision, funding acquisition, formal analysis, writing – review and editing.

## Conflicts of Interest

The authors declare no conflicts of interest.

## Supporting information


Supporting File 1

Supporting File 2

## Data Availability

The data that support the findings of this study are available in the supporting information of this article.
