# Supplementary material for: Clinical Manifestations Associated With RT‐PCR–Confirmed Zika Virus Infection Among Adults With Acute Rash in the Brazilian Amazon: A Cross‐Sectional Study
Source: J Med Virol. 2026 Sep 7;98(9):e71109. doi: 10.1002/jmv.71109 (PMC13548340; doi:10.1002/jmv.71109)
Supplement: Supplementary file 1 [file JMV-98-e71109-s002.docx]

**Supplementary Table 1. Annual distribution of RT-qPCR-confirmed and non-confirmed ZIKV cases among adults presenting with acute rash at FMT-HVD, Manaus, Brazil, from March 2017 to December 2022.**

| **Year** | **Detected cases** | **Zika prevalence by PCR**  **%** | **95% CI^1^** |
| --- | --- | --- | --- |
| 2017 (n=124) | 51 | 41.1 | 32.5-49.8 |
| 2018 (n=425) | 227 | 53.4 | 48.7-58.1 |
| 2019 (n=75) | 41 | 54.7 | 43.4-65.9 |
| 2021 (n=69) | 0 | 0 | 0 |
| 2022 (n=431) | 0 | 0 | 0 |
| Total (n=1,124) | 319 | 28.4 | 25.7 – 31.0 |

^1^ 95% confidence interval.

No participants were enrolled in 2020 due to interruption/reorganization of study activities. The absence of confirmed cases in 2021 and 2022 may reflect temporal variation in local transmission and/or differences in the screened population and should be interpreted accordingly.

**Supplementary Table 2. Clinical manifestations according to RT-qPCR-confirmed ZIKV infection status among adults presenting with acute rash.**

| Signs and symptoms | Zika positive  n (%) | Zika negative n (%) | Total  n (%) | p-value^2^ |
| --- | --- | --- | --- | --- |
|  | n = 319^1^ | n = 805^1^ | n = 1,124^1^ |  |
| Fever | 265/318 (83%) | 749/802 (93%) | 1,014/1,120 (91%) | <0.001 |
| Pruritus | 300/319 (94%) | 365/802 (46%) | 665/1,121 (59%) | <0.001 |
| Conjunctivitis | 231/317 (73%) | 212/800 (27%) | 443/1,117 (40%) | <0.001 |
| Arthralgia | 258/319 (81%) | 513/802 (64%) | 771/1,121 (69%) | <0.001 |
| Edema | 216/316 (68%) | 235/798 (29%) | 451/1,114 (40%) | <0.001 |
| Headache | 24/33 (73%) | 476/505 (94%) | 500/538 (93%) | <0.001 |
| Myalgia | 23/33 (70%) | 438/506 (87%) | 461/539 (86%) | 0.017 |
| Lymphadenopathy | 10/31 (32%) | 1/6 (17%) | 11/37 (30%) | 0.6 |
| Diarrhea | 7/32 (22%) | 128/503 (25%) | 135/535 (25%) | 0.7 |
| Nausea | 15/33 (45%) | 6/9 (67%) | 21/42 (50%) | 0.5 |
| Vomiting | 6/33 (18%) | 125/506 (25%) | 131/539 (24%) | 0.4 |
| Retro-orbital pain | 3/5 (60%) | 224/500 (45%) | 227/505 (45%) | 0.7 |
| Bleeding | 1/1 (100%) | 25/494 (5.1%) | 26/495 (5.3%) | 0.053 |
| Fever and Pruritus | 252/318 (79%) | 327/802 (41%) | 579/1,120 (52%) | <0.001 |
| Fever and conjunctivitis | 194/317 (61%) | 196/800 (25%) | 390/1,117 (35%) | <0.001 |
| Fever and arthralgia | 216/318 (68%) | 481/802 (60%) | 697/1,120 (62%) | 0.013 |
| Fever and edema | 182/315 (58%) | 212/798 (27%) | 394/1,113 (35%) | <0.001 |
| Fever and headache | 20/79 (25%) | 468/546 (86%) | 488/625 (78%) | <0.001 |
| Fever and myalgia | 17/78 (22%) | 429/547 (78%) | 446/625 (71%) | <0.001 |
| Fever and lymphadenopathy | 10/77 (13%) | 1/58 (1.7%) | 11/135 (8.1%) | 0.024 |
| Fever and diarrhea | 6/77 (7.8%) | 125/544 (23%) | 131/621 (21%) | 0.002 |
| Fever and nausea | 13/79 (16%) | 6/61 (9.8%) | 19/140 (14%) | 0.3 |
| Fever and vomiting | 6/79 (7.6%) | 123/547 (22%) | 129/626 (21%) | 0.002 |
| Fever and Retro-orbital pain | 2/56 (3.6%) | 220/542 (41%) | 222/598 (37%) | <0.001 |
| Fever and bleeding | 1/54 (1.9%) | 25/536 (4.7%) | 26/590 (4.4%) | 0.5 |
| Pruritus and conjunctivitis | 217/317 (68%) | 164/802 (20%) | 381/1,119 (34%) | <0.001 |
| Pruritus and arthralgia | 246/319 (77%) | 272/802 (34%) | 518/1,121 (46%) | <0.001 |
| Pruritus and edema | 206/316 (65%) | 189/800 (24%) | 395/1,116 (35%) | <0.001 |
| Pruritus and headache | 22/49 (45%) | 112/564 (20%) | 134/613 (22%) | <0.001 |
| Pruritus and myalgia | 22/49 (45%) | 115/564 (20%) | 137/613 (22%) | <0.001 |
| Pruritus and lymphadenopathy | 10/47 (21%) | 1/441 (0.2%) | 11/488 (2.3%) | <0.001 |
| Pruritus and diarrhea | 6/48 (13%) | 41/563 (7.3%) | 47/611 (7.7%) | 0.3 |
| Pruritus and nausea | 14/49 (29%) | 3/442 (0.7%) | 17/491 (3.5%) | <0.001 |
| Pruritus and vomiting | 5/49 (10%) | 36/564 (6.4%) | 41/613 (6.7%) | 0.4 |
| Pruritus and Retro-orbital pain | 1/22 (4.5%) | 86/560 (15%) | 87/582 (15%) | 0.2 |
| Pruritus and bleeding | 1/20 (5.0%) | 17/559 (3.0%) | 18/579 (3.1%) | 0.5 |
| Conjunctivitis and arthralgia | 192/317 (61%) | 177/801 (22%) | 369/1,118 (33%) | <0.001 |
| Conjunctivitis and edema | 169/316 (53%) | 120/799 (15%) | 289/1,115 (26%) | <0.001 |
| Conjunctivitis and headache | 15/107 (14%) | 62/658 (9.4%) | 77/765 (10%) | 0.14 |
| Conjunctivitis and myalgia | 13/107 (12%) | 61/657 (9.3%) | 74/764 (9.7%) | 0.4 |
| Conjunctivitis and lymphadenopathy | 10/108 (9.3%) | 1/592 (0.2%) | 11/700 (1.6%) | <0.001 |
| Conjunctivitis and diarrhea | 4/108 (3.7%) | 25/657 (3.8%) | 29/765 (3.8%) | >0.9 |
| Conjunctivitis and nausea | 10/108 (9.3%) | 4/593 (0.7%) | 14/701 (2.0%) | <0.001 |
| Conjunctivitis and vomiting | 4/109 (3.7%) | 16/659 (2.4%) | 20/768 (2.6%) | 0.5 |
| Conjunctivitis and Retro-orbital pain | 2/90 (2.2%) | 52/654 (8.0%) | 54/744 (7.3%) | 0.05 |
| Conjunctivitis and bleeding | 1/87 (1.1%) | 6/654 (0.9%) | 7/741 (0.9%) | 0.6 |
| Arthralgia and edema | 192/317 (61%) | 194/800 (24%) | 386/1,117 (35%) | <0.001 |
| Arthralgia and headache | 18/87 (21%) | 291/596 (49%) | 309/683 (45%) | <0.001 |
| Arthralgia and myalgia | 18/87 (21%) | 284/596 (48%) | 302/683 (44%) | <0.001 |
| Arthralgia and lymphadenopathy | 10/86 (12%) | 1/294 (0.3%) | 11/380 (2.9%) | <0.001 |
| Arthralgia and diarrhea | 6/87 (6.9%) | 85/594 (14%) | 91/681 (13%) | 0.058 |
| Arthralgia and nausea | 12/87 (14%) | 5/296 (1.7%) | 17/383 (4.4%) | <0.001 |
| Arthralgia and vomiting | 4/87 (4.6%) | 80/596 (13%) | 84/683 (12%) | 0.019 |
| Arthralgia and Retro-orbital pain | 3/64 (4.7%) | 190/591 (32%) | 193/655 (29%) | <0.001 |
| Arthralgia and bleeding | 1/62 (1.6%) | 23/587 (3.9%) | 24/649 (3.7%) | 0.7 |
| Edema and headache | 10/116 (8.6%) | 58/633 (9.2%) | 68/749 (9.1%) | 0.9 |
| Edema and myalgia | 10/116 (8.6%) | 64/633 (10%) | 74/749 (9.9%) | 0.6 |
| Edema and lymphadenopathy | 7/117 (6.0%) | 1/567 (0.2%) | 8/684 (1.2%) | <0.001 |
| Edema and diarrhea | 6/118 (5.1%) | 22/633 (3.5%) | 28/751 (3.7%) | 0.4 |
| Edema and nausea | 8/117 (6.8%) | 3/567 (0.5%) | 11/684 (1.6%) | <0.001 |
| Edema and vomiting | 4/118 (3.4%) | 18/635 (2.8%) | 22/753 (2.9%) | 0.8 |
| Edema and Retro-orbital pain | 2/103 (1.9%) | 46/630 (7.3%) | 48/733 (6.5%) | 0.041 |
| Edema and bleeding | 0/100 (0%) | 9/629 (1.4%) | 9/729 (1.2%) | 0.6 |
| Headache and myalgia | 18/33 (55%) | 417/505 (83%) | 435/538 (81%) | <0.001 |
| Headache and lymphadenopathy | 8/31 (26%) | 1/33 (3.0%) | 9/64 (14%) | 0.012 |
| Headache and diarrhea | 6/32 (19%) | 123/503 (24%) | 129/535 (24%) | 0.5 |
| Headache and nausea | 13/33 (39%) | 6/36 (17%) | 19/69 (28%) | 0.035 |
| Headache and vomiting | 6/33 (18%) | 119/506 (24%) | 125/539 (23%) | 0.5 |
| Headache andretro-orbital pain | 2/13 (15%) | 213/502 (42%) | 215/515 (42%) | 0.051 |
| Headache and bleeding | 1/10 (10%) | 22/496 (4.4%) | 23/506 (4.5%) | 0.4 |
| Myalgia and lymphadenopathy | 6/31 (19%) | 1/73 (1.4%) | 7/104 (6.7%) | 0.003 |
| Myalgia and diarrhea | 5/32 (16%) | 116/504 (23%) | 121/536 (23%) | 0.3 |
| Myalgia and nausea | 11/33 (33%) | 5/75 (6.7%) | 16/108 (15%) | <0.001 |
| Myalgia and vomiting | 5/33 (15%) | 116/506 (23%) | 121/539 (22%) | 0.3 |
| Myalgia and Retro-orbital pain | 1/13 (7.7%) | 204/501 (41%) | 205/514 (40%) | 0.016 |
| Myalgia and bleeding | 0/10 (0%) | 24/497 (4.8%) | 24/507 (4.7%) | >0.9 |
| Lymphadenopathy and diarrhea | 3/31 (9.7%) | 0/376 (0%) | 3/407 (0.7%) | <0.001 |
| Lymphadenopathy and nausea | 5/31 (16%) | 1/6 (17%) | 6/37 (16%) | >0.9 |
| Lymphadenopathy and vomiting | 2/31 (6.5%) | 0/382 (0%) | 2/413 (0.5%) | 0.005 |
| Lymphadenopathy and bleeding | 1/22 (4.5%) | 0/474 (0%) | 1/496 (0.2%) | 0.044 |
| Diarrhea and nausea | 5/32 (16%) | 1/376 (0.3%) | 6/408 (1.5%) | <0.001 |
| Diarrhea and vomiting | 5/32 (16%) | 61/504 (12%) | 66/536 (12%) | 0.6 |
| Diarrhea and Retro-orbital pain | 0/26 (0%) | 69/502 (14%) | 69/528 (13%) | 0.036 |
| Diarrhea and bleeding | 0/25 (0%) | 8/499 (1.6%) | 8/524 (1.5%) | >0.9 |
| Nausea and vomiting | 6/33 (18%) | 3/384 (0.8%) | 9/417 (2.2%) | <0.001 |
| Nausea and Retro-orbital pain | 1/20 (5.0%) | 3/282 (1.1%) | 4/302 (1.3%) | 0.2 |
| Vomiting and Retro-orbital pain | 0/28 (0%) | 65/505 (13%) | 65/533 (12%) | 0.037 |
| Vomiting and bleeding | 0/27 (0%) | 11/503 (2.2%) | 11/530 (2.1%) | >0.9 |
| Retro-orbital pain Arthralgia and and bleeding | 0/2 (0%) | 20/497 (4.0%) | 20/499 (4.0%) | >0.9 |
| Fever and Pruritus and and conjunctivitis | 185/317 (58%) | 151/802 (19%) | 336/1,119 (30%) | <0.001 |
| Fever and Pruritus and and Arthralgia | 209/318 (66%) | 251/802 (31%) | 460/1,120 (41%) | <0.001 |
| Fever and Pruritus and and edema | 176/315 (56%) | 171/800 (21%) | 347/1,115 (31%) | <0.001 |
| Fever and Pruritus and and headache | 18/90 (20%) | 108/594 (18%) | 126/684 (18%) | 0.7 |
| Fever and Pruritus and and Myalgia | 16/89 (18%) | 110/594 (19%) | 126/683 (18%) | >0.9 |
| Fever and Pruritus and and lymphadenopathy | 10/88 (11%) | 1/478 (0.2%) | 11/566 (1.9%) | <0.001 |
| Fever and Pruritus and and diarrhea | 5/88 (5.7%) | 40/593 (6.7%) | 45/681 (6.6%) | 0.7 |
| Fever and Pruritus and and nausea | 12/90 (13%) | 3/479 (0.6%) | 15/569 (2.6%) | <0.001 |
| Fever and Pruritus and and vomiting | 5/90 (5.6%) | 36/594 (6.1%) | 41/684 (6.0%) | 0.9 |
| Fever and pruritus and Retro-orbital pain | 1/68 (1.5%) | 84/591 (14%) | 85/659 (13%) | 0.003 |
| Fever and Pruritus and and bleeding | 1/67 (1.5%) | 17/590 (2.9%) | 18/657 (2.7%) | >0.9 |
| Fever and conjunctivitis and and arthralgia | 164/317 (52%) | 163/801 (20%) | 327/1,118 (29%) | <0.001 |
| Fever and conjunctivitis and and edema | 145/316 (46%) | 112/799 (14%) | 257/1,115 (23%) | <0.001 |
| Fever and conjunctivitis and and headache | 13/139 (9.4%) | 61/670 (9.1%) | 74/809 (9.1%) | >0.9 |
| Fever and conjunctivitis and and myalgia | 10/139 (7.2%) | 59/669 (8.8%) | 69/808 (8.5%) | 0.5 |
| Fever and conjunctivitis and and lymphadenopathy | 10/140 (7.1%) | 1/608 (0.2%) | 11/748 (1.5%) | <0.001 |
| Fever and conjunctivitis and and diarrhea | 4/140 (2.9%) | 25/669 (3.7%) | 29/809 (3.6%) | 0.6 |
| Fever and conjunctivitis and and nausea | 9/140 (6.4%) | 4/609 (0.7%) | 13/749 (1.7%) | <0.001 |
| Fever and conjunctivitis and and vomiting | 4/141 (2.8%) | 16/671 (2.4%) | 20/812 (2.5%) | 0.8 |
| Fever and conjunctivitis and Retro-orbital pain | 1/125 (0.8%) | 50/666 (7.5%) | 51/791 (6.4%) | 0.005 |
| Fever and conjunctivitis and and bleeding | 1/124 (0.8%) | 6/666 (0.9%) | 7/790 (0.9%) | >0.9 |
| Fever and arthralgia and and edema | 162/316 (51%) | 176/800 (22%) | 338/1,116 (30%) | <0.001 |
| Fever and Arthralgia and and headache | 16/123 (13%) | 285/618 (46%) | 301/741 (41%) | <0.001 |
| Fever and arthralgia and and myalgia | 14/122 (11%) | 277/618 (45%) | 291/740 (39%) | <0.001 |
| Fever and arthralgia and and lymphadenopathy | 10/122 (8.2%) | 1/325 (0.3%) | 11/447 (2.5%) | <0.001 |
| Fever and Arthralgia and and diarrhea | 5/122 (4.1%) | 83/616 (13%) | 88/738 (12%) | 0.004 |
| Fever and arthralgia and nausea | 10/123 (8.1%) | 5/327 (1.5%) | 15/450 (3.3%) | 0.001 |
| Fever and arthralgia and and vomiting | 4/123 (3.3%) | 79/618 (13%) | 83/741 (11%) | 0.002 |
| Fever and arthralgia and Retro-orbital pain | 2/104 (1.9%) | 186/614 (30%) | 188/718 (26%) | <0.001 |
| Fever and arthralgia and and bleeding | 1/103 (1.0%) | 23/610 (3.8%) | 24/713 (3.4%) | 0.2 |
| Fever and edema and and headache | 9/146 (6.2%) | 54/649 (8.3%) | 63/795 (7.9%) | 0.4 |
| Fever and edema and and myalgia | 8/145 (5.5%) | 59/649 (9.1%) | 67/794 (8.4%) | 0.2 |
| Fever and edema and lymphadenopathy | 7/147 (4.8%) | 1/590 (0.2%) | 8/737 (1.1%) | <0.001 |
| Fever and edema and and diarrhea | 5/147 (3.4%) | 21/649 (3.2%) | 26/796 (3.3%) | >0.9 |
| Fever and edema and and nausea | 8/147 (5.4%) | 3/590 (0.5%) | 11/737 (1.5%) | <0.001 |
| Fever and edema and and vomiting | 4/148 (2.7%) | 18/651 (2.8%) | 22/799 (2.8%) | >0.9 |
| Fever and edema and Retro-orbital pain | 1/135 (0.7%) | 44/646 (6.8%) | 45/781 (5.8%) | 0.006 |
| Fever and edema and and bleeding | 0/133 (0%) | 9/645 (1.4%) | 9/778 (1.2%) | 0.4 |
| Fever and headache and and Myalgia | 14/79 (18%) | 409/546 (75%) | 423/625 (68%) | <0.001 |
| Fever and headache and and lymphadenopathy | 8/77 (10%) | 1/82 (1.2%) | 9/159 (5.7%) | 0.015 |
| Fever and headache and and diarrhea | 6/78 (7.7%) | 120/544 (22%) | 126/622 (20%) | 0.003 |
| Fever and headache and and nausea | 12/79 (15%) | 6/85 (7.1%) | 18/164 (11%) | 0.1 |
| Fever and headache and and vomiting | 6/79 (7.6%) | 117/547 (21%) | 123/626 (20%) | 0.004 |
| Fever and headache andretro-orbital pain pain | 2/62 (3.2%) | 211/543 (39%) | 213/605 (35%) | <0.001 |
| Fever and headache and and bleeding | 1/60 (1.7%) | 22/537 (4.1%) | 23/597 (3.9%) | 0.7 |
| Fever and myalgia and lymphadenopathy | 6/77 (7.8%) | 1/123 (0.8%) | 7/200 (3.5%) | 0.014 |
| Fever and Myalgia and and diarrhea | 4/77 (5.2%) | 113/545 (21%) | 117/622 (19%) | 0.001 |
| Fever and Myalgia and and nausea | 9/79 (11%) | 5/125 (4.0%) | 14/204 (6.9%) | 0.042 |
| Fever and Myalgia and and vomiting | 5/79 (6.3%) | 114/547 (21%) | 119/626 (19%) | 0.002 |
| Fever and myalgia and Retro-orbital pain | 1/63 (1.6%) | 202/542 (37%) | 203/605 (34%) | <0.001 |
| Fever and Myalgia and and bleeding | 0/61 (0%) | 24/538 (4.5%) | 24/599 (4.0%) | 0.2 |
| Fever and lymphadenopathy and and diarrhea | 3/77 (3.9%) | 0/420 (0%) | 3/497 (0.6%) | 0.004 |
| Fever and lymphadenopathy and nausea | 5/77 (6.5%) | 1/58 (1.7%) | 6/135 (4.4%) | 0.2 |
| Fever and lymphadenopathy and vomiting | 2/77 (2.6%) | 0/425 (0%) | 2/502 (0.4%) | 0.023 |
| Fever and lymphadenopathy and and bleeding | 1/68 (1.5%) | 0/515 (0%) | 1/583 (0.2%) | 0.12 |
| Fever and diarrhea and and nausea | 5/78 (6.4%) | 1/420 (0.2%) | 6/498 (1.2%) | <0.001 |
| Fever and diarrhea and and vomiting | 5/78 (6.4%) | 59/545 (11%) | 64/623 (10%) | 0.2 |
| Fever and diarrhea and Retro-orbital pain | 0/72 (0%) | 68/543 (13%) | 68/615 (11%) | 0.001 |
| Fever and diarrhea and and bleeding | 0/71 (0%) | 8/540 (1.5%) | 8/611 (1.3%) | 0.6 |
| Fever and nausea and and vomiting | 6/79 (7.6%) | 3/427 (0.7%) | 9/506 (1.8%) | <0.001 |
| Fever and nausea and Retro-orbital pain | 1/68 (1.5%) | 3/327 (0.9%) | 4/395 (1.0%) | 0.5 |
| Fever and vomiting and Retro-orbital pain | 0/74 (0%) | 64/546 (12%) | 64/620 (10%) | 0.002 |
| Fever and vomiting and and bleeding | 0/73 (0%) | 11/544 (2.0%) | 11/617 (1.8%) | 0.4 |
| Fever and Retro-orbital pain bleeding | 0/54 (0%) | 20/539 (3.7%) | 20/593 (3.4%) | 0.2 |
| Pruritus and conjunctivitis and and arthralgia | 183/317 (58%) | 137/802 (17%) | 320/1,119 (29%) | <0.001 |
| Pruritus and conjunctivitis and and edema | 159/316 (50%) | 103/800 (13%) | 262/1,116 (23%) | <0.001 |
| Pruritus and conjunctivitis and and headache | 15/120 (13%) | 39/684 (5.7%) | 54/804 (6.7%) | 0.006 |
| Pruritus and conjunctivitis and and myalgia | 13/120 (11%) | 41/684 (6.0%) | 54/804 (6.7%) | 0.051 |
| Pruritus and conjunctivitis and and lymphadenopathy | 10/121 (8.3%) | 1/640 (0.2%) | 11/761 (1.4%) | <0.001 |
| Pruritus and conjunctivitis and and diarrhea | 4/121 (3.3%) | 15/683 (2.2%) | 19/804 (2.4%) | 0.5 |
| Pruritus and conjunctivitis and and nausea | 10/121 (8.3%) | 3/641 (0.5%) | 13/762 (1.7%) | <0.001 |
| Pruritus and conjunctivitis and and vomiting | 4/122 (3.3%) | 9/684 (1.3%) | 13/806 (1.6%) | 0.12 |
| Pruritus and conjunctivitis andretro-orbital pain | 1/103 (1.0%) | 34/682 (5.0%) | 35/785 (4.5%) | 0.072 |
| Pruritus and conjunctivitis and and bleeding | 1/101 (1.0%) | 6/681 (0.9%) | 7/782 (0.9%) | >0.9 |
| Pruritus and Arthralgia and and edema | 184/317 (58%) | 158/801 (20%) | 342/1,118 (31%) | <0.001 |
| Pruritus and Arthralgia and and headache | 17/97 (18%) | 93/634 (15%) | 110/731 (15%) | 0.5 |
| Pruritus and arthralgia and and myalgia | 17/97 (18%) | 98/634 (15%) | 115/731 (16%) | 0.6 |
| Pruritus and arthralgia and and lymphadenopathy | 10/96 (10%) | 1/534 (0.2%) | 11/630 (1.7%) | <0.001 |
| Pruritus and Arthralgia and and diarrhea | 6/97 (6.2%) | 38/634 (6.0%) | 44/731 (6.0%) | >0.9 |
| Pruritus and arthralgia and nausea | 12/97 (12%) | 2/534 (0.4%) | 14/631 (2.2%) | <0.001 |
| Pruritus and Arthralgia and and vomiting | 4/97 (4.1%) | 29/634 (4.6%) | 33/731 (4.5%) | >0.9 |
| Pruritus and arthralgia andretro-orbital pain pain | 1/74 (1.4%) | 79/630 (13%) | 80/704 (11%) | 0.004 |
| Pruritus and Arthralgia and and bleeding | 1/74 (1.4%) | 17/630 (2.7%) | 18/704 (2.6%) | 0.7 |
| Pruritus and Edema and and headache | 10/125 (8.0%) | 42/662 (6.3%) | 52/787 (6.6%) | 0.5 |
| Pruritus and edema and and myalgia | 10/125 (8.0%) | 47/663 (7.1%) | 57/788 (7.2%) | 0.7 |
| Pruritus and edema and lymphadenopathy | 7/126 (5.6%) | 1/613 (0.2%) | 8/739 (1.1%) | <0.001 |
| Pruritus and Edema and and diarrhea | 6/127 (4.7%) | 18/663 (2.7%) | 24/790 (3.0%) | 0.3 |
| Pruritus and Edema and and nausea | 8/126 (6.3%) | 1/612 (0.2%) | 9/738 (1.2%) | <0.001 |
| Pruritus and Edema and and vomiting | 4/127 (3.1%) | 14/664 (2.1%) | 18/791 (2.3%) | 0.5 |
| Pruritus and edema and Retro-orbital pain | 1/112 (0.9%) | 36/661 (5.4%) | 37/773 (4.8%) | 0.037 |
| Pruritus and Edema and and bleeding | 0/110 (0%) | 8/661 (1.2%) | 8/771 (1.0%) | 0.6 |
| Pruritus and headache and and Myalgia | 17/49 (35%) | 106/564 (19%) | 123/613 (20%) | 0.008 |
| Pruritus and headache and and lymphadenopathy | 8/47 (17%) | 1/455 (0.2%) | 9/502 (1.8%) | <0.001 |
| Pruritus and headache and and diarrhea | 5/48 (10%) | 38/563 (6.7%) | 43/611 (7.0%) | 0.4 |
| Pruritus and headache and and nausea | 12/49 (24%) | 3/456 (0.7%) | 15/505 (3.0%) | <0.001 |
| Pruritus and headache and and vomiting | 5/49 (10%) | 31/564 (5.5%) | 36/613 (5.9%) | 0.2 |
| Pruritus and headache andretro-orbital pain pain | 1/30 (3.3%) | 76/561 (14%) | 77/591 (13%) | 0.2 |
| Pruritus and headache and and bleeding | 1/28 (3.6%) | 14/560 (2.5%) | 15/588 (2.6%) | 0.5 |
| Pruritus and myalgia and lymphadenopathy | 6/47 (13%) | 1/452 (0.2%) | 7/499 (1.4%) | <0.001 |
| Pruritus and Myalgia and and diarrhea | 5/48 (10%) | 39/564 (6.9%) | 44/612 (7.2%) | 0.4 |
| Pruritus and myalgia and nausea | 11/49 (22%) | 2/452 (0.4%) | 13/501 (2.6%) | <0.001 |
| Pruritus and Myalgia and and vomiting | 5/49 (10%) | 35/564 (6.2%) | 40/613 (6.5%) | 0.2 |
| Pruritus and myalgia and Retro-orbital pain | 0/29 (0%) | 79/561 (14%) | 79/590 (13%) | 0.023 |
| Pruritus and Myalgia and and bleeding | 0/27 (0%) | 17/561 (3.0%) | 17/588 (2.9%) | >0.9 |
| Pruritus and lymphadenopathy and and diarrhea | 3/47 (6.4%) | 0/522 (0%) | 3/569 (0.5%) | <0.001 |
| Pruritus and lymphadenopathy and nausea | 5/47 (11%) | 1/441 (0.2%) | 6/488 (1.2%) | <0.001 |
| Pruritus and lymphadenopathy and and vomiting | 2/47 (4.3%) | 0/528 (0%) | 2/575 (0.3%) | 0.007 |
| Pruritus and lymphadenopathy and and bleeding | 1/38 (2.6%) | 0/545 (0%) | 1/583 (0.2%) | 0.065 |
| Pruritus and diarrhea and and nausea | 4/48 (8.3%) | 0/522 (0%) | 4/570 (0.7%) | <0.001 |
| Pruritus and diarrhea and and vomiting | 4/48 (8.3%) | 17/564 (3.0%) | 21/612 (3.4%) | 0.074 |
| Pruritus and diarrhea and Retro-orbital pain | 0/43 (0%) | 33/563 (5.9%) | 33/606 (5.4%) | 0.2 |
| Pruritus and diarrhea and and bleeding | 0/42 (0%) | 6/563 (1.1%) | 6/605 (1.0%) | >0.9 |
| Pruritus and nausea and vomiting | 5/49 (10%) | 0/528 (0%) | 5/577 (0.9%) | <0.001 |
| Pruritus and nausea and Retro-orbital pain | 1/37 (2.7%) | 1/477 (0.2%) | 2/514 (0.4%) | 0.14 |
| Pruritus and vomiting andretro-orbital pain | 0/45 (0%) | 24/564 (4.3%) | 24/609 (3.9%) | 0.2 |
| Pruritus and vomiting and and bleeding | 0/44 (0%) | 8/564 (1.4%) | 8/608 (1.3%) | >0.9 |
| Pruritus andretro-orbital pain bleeding | 0/21 (0%) | 15/559 (2.7%) | 15/580 (2.6%) | >0.9 |
| Conjunctivitis and arthralgia and and edema | 154/317 (49%) | 110/801 (14%) | 264/1,118 (24%) | <0.001 |
| Conjunctivitis and arthralgia and and headache | 11/141 (7.8%) | 51/681 (7.5%) | 62/822 (7.5%) | 0.9 |
| Conjunctivitis and arthralgia and myalgia | 9/141 (6.4%) | 51/681 (7.5%) | 60/822 (7.3%) | 0.6 |
| Conjunctivitis and arthralgia and and lymphadenopathy | 10/143 (7.0%) | 1/627 (0.2%) | 11/770 (1.4%) | <0.001 |
| Conjunctivitis and arthralgia and and diarrhea | 4/143 (2.8%) | 20/681 (2.9%) | 24/824 (2.9%) | >0.9 |
| Conjunctivitis and arthralgia and and nausea | 9/142 (6.3%) | 3/627 (0.5%) | 12/769 (1.6%) | <0.001 |
| Conjunctivitis and arthralgia and and vomiting | 3/143 (2.1%) | 16/682 (2.3%) | 19/825 (2.3%) | >0.9 |
| Conjunctivitis and arthralgia and pain and retroorbital | 2/127 (1.6%) | 44/678 (6.5%) | 46/805 (5.7%) | 0.029 |
| Conjunctivitis and arthralgia and and bleeding | 1/126 (0.8%) | 6/679 (0.9%) | 7/805 (0.9%) | >0.9 |
| Conjunctivitis and edema and and headache | 8/159 (5.0%) | 26/711 (3.7%) | 34/870 (3.9%) | 0.4 |
| Conjunctivitis and edema and myalgia | 7/159 (4.4%) | 27/711 (3.8%) | 34/870 (3.9%) | 0.7 |
| Conjunctivitis and edema and and lymphadenopathy | 7/161 (4.3%) | 1/683 (0.1%) | 8/844 (0.9%) | <0.001 |
| Conjunctivitis and edema and and diarrhea | 4/161 (2.5%) | 12/712 (1.7%) | 16/873 (1.8%) | 0.5 |
| Conjunctivitis and edema and nausea | 7/160 (4.4%) | 2/682 (0.3%) | 9/842 (1.1%) | <0.001 |
| Conjunctivitis and edema and and vomiting | 3/161 (1.9%) | 7/713 (1.0%) | 10/874 (1.1%) | 0.4 |
| Conjunctivitis and edema and Retro-orbital pain | 2/150 (1.3%) | 23/708 (3.2%) | 25/858 (2.9%) | 0.3 |
| Conjunctivitis and edema and bleeding | 0/147 (0%) | 4/708 (0.6%) | 4/855 (0.5%) | >0.9 |
| Conjunctivitis and headache and myalgia | 10/107 (9.3%) | 56/658 (8.5%) | 66/765 (8.6%) | 0.8 |
| Conjunctivitis and headache and and lymphadenopathy | 8/108 (7.4%) | 1/599 (0.2%) | 9/707 (1.3%) | <0.001 |
| Conjunctivitis and headache and and diarrhea | 4/108 (3.7%) | 24/657 (3.7%) | 28/765 (3.7%) | >0.9 |
| Conjunctivitis and headache and and nausea | 8/108 (7.4%) | 4/600 (0.7%) | 12/708 (1.7%) | <0.001 |
| Conjunctivitis and headache and and vomiting | 4/109 (3.7%) | 14/659 (2.1%) | 18/768 (2.3%) | 0.3 |
| Conjunctivitis and headache andretro-orbital pain | 1/95 (1.1%) | 47/655 (7.2%) | 48/750 (6.4%) | 0.023 |
| Conjunctivitis and headache and and bleeding | 1/93 (1.1%) | 4/655 (0.6%) | 5/748 (0.7%) | 0.5 |
| Conjunctivitis and myalgia and and lymphadenopathy | 6/108 (5.6%) | 1/600 (0.2%) | 7/708 (1.0%) | <0.001 |
| Conjunctivitis and myalgia and and diarrhea | 3/108 (2.8%) | 23/658 (3.5%) | 26/766 (3.4%) | >0.9 |
| Conjunctivitis and myalgia and nausea | 7/108 (6.5%) | 3/600 (0.5%) | 10/708 (1.4%) | <0.001 |
| Conjunctivitis and myalgia and and vomiting | 4/109 (3.7%) | 16/659 (2.4%) | 20/768 (2.6%) | 0.5 |
| Conjunctivitis and myalgia and Retro-orbital pain | 0/96 (0%) | 44/654 (6.7%) | 44/750 (5.9%) | 0.009 |
| Conjunctivitis and myalgia and and bleeding | 0/94 (0%) | 6/655 (0.9%) | 6/749 (0.8%) | >0.9 |
| Conjunctivitis and lymphadenopathy and and diarrhea | 3/108 (2.8%) | 0/633 (0%) | 3/741 (0.4%) | 0.003 |
| Conjunctivitis and lymphadenopathy and nausea | 5/108 (4.6%) | 1/592 (0.2%) | 6/700 (0.9%) | <0.001 |
| Conjunctivitis and lymphadenopathy and and vomiting | 2/108 (1.9%) | 0/644 (0%) | 2/752 (0.3%) | 0.02 |
| Conjunctivitis and lymphadenopathy and and bleeding | 1/99 (1.0%) | 0/651 (0%) | 1/750 (0.1%) | 0.13 |
| Conjunctivitis and diarrhea and and nausea | 3/108 (2.8%) | 1/633 (0.2%) | 4/741 (0.5%) | 0.011 |
| Conjunctivitis and diarrhea and and vomiting | 3/108 (2.8%) | 7/659 (1.1%) | 10/767 (1.3%) | 0.2 |
| Conjunctivitis and diarrhea and Retro-orbital pain | 0/105 (0%) | 22/656 (3.4%) | 22/761 (2.9%) | 0.059 |
| Conjunctivitis and diarrhea and and bleeding | 0/104 (0%) | 3/657 (0.5%) | 3/761 (0.4%) | >0.9 |
| Conjunctivitis and nausea and vomiting | 4/109 (3.7%) | 1/644 (0.2%) | 5/753 (0.7%) | 0.002 |
| Conjunctivitis and nausea and Retro-orbital pain | 1/100 (1.0%) | 1/604 (0.2%) | 2/704 (0.3%) | 0.3 |
| Conjunctivitis and vomiting and Retro-orbital pain | 0/106 (0%) | 11/658 (1.7%) | 11/764 (1.4%) | 0.4 |
| Conjunctivitis and vomiting and and bleeding | 0/105 (0%) | 2/658 (0.3%) | 2/763 (0.3%) | >0.9 |
| Conjunctivitis and Retro-orbital pain bleeding | 0/88 (0%) | 5/654 (0.8%) | 5/742 (0.7%) | >0.9 |
| Arthralgia and edema and and headache. | 10/141 (7.1%) | 53/668 (7.9%) | 63/809 (7.8%) | 0.7 |
| Arthralgia and edema and myalgia | 10/141 (7.1%) | 58/668 (8.7%) | 68/809 (8.4%) | 0.5 |
| Arthralgia and edema and lymphadenopathy | 7/142 (4.9%) | 1/609 (0.2%) | 8/751 (1.1%) | <0.001 |
| Arthralgia and edema and and diarrhea | 6/143 (4.2%) | 22/668 (3.3%) | 28/811 (3.5%) | 0.6 |
| Arthralgia and edema and nausea | 8/142 (5.6%) | 3/609 (0.5%) | 11/751 (1.5%) | <0.001 |
| Arthralgia and edema and vomiting | 4/143 (2.8%) | 14/669 (2.1%) | 18/812 (2.2%) | 0.5 |
| Arthralgia and edema and Retro-orbital pain | 2/127 (1.6%) | 43/666 (6.5%) | 45/793 (5.7%) | 0.029 |
| Arthralgia and edema and and bleeding | 0/125 (0%) | 9/665 (1.4%) | 9/790 (1.1%) | 0.4 |
| Arthralgia and headache and and myalgia | 13/87 (15%) | 271/596 (45%) | 284/683 (42%) | <0.001 |
| Arthralgia and headache and and lymphadenopathy | 8/86 (9.3%) | 1/309 (0.3%) | 9/395 (2.3%) | <0.001 |
| Arthralgia and headache and and diarrhea. | 5/87 (5.7%) | 82/594 (14%) | 87/681 (13%) | 0.035 |
| Arthralgia and headache and and nausea | 10/87 (11%) | 5/311 (1.6%) | 15/398 (3.8%) | <0.001 |
| Arthralgia and headache and and vomiting | 4/87 (4.6%) | 76/596 (13%) | 80/683 (12%) | 0.027 |
| Arthralgia and headache andretro-orbital pain | 2/71 (2.8%) | 180/592 (30%) | 182/663 (27%) | <0.001 |
| Arthralgia and headache and and bleeding. | 1/70 (1.4%) | 20/588 (3.4%) | 21/658 (3.2%) | 0.7 |
| Arthralgia and myalgia and lymphadenopathy | 6/86 (7.0%) | 1/316 (0.3%) | 7/402 (1.7%) | <0.001 |
| Arthralgia and myalgia and and diarrhea | 5/87 (5.7%) | 82/594 (14%) | 87/681 (13%) | 0.035 |
| Arthralgia and myalgia and nausea | 9/87 (10%) | 5/318 (1.6%) | 14/405 (3.5%) | <0.001 |
| Arthralgia and myalgia and vomiting | 4/87 (4.6%) | 78/596 (13%) | 82/683 (12%) | 0.023 |
| Arthralgia and myalgia and Retro-orbital pain | 1/70 (1.4%) | 179/592 (30%) | 180/662 (27%) | <0.001 |
| Arthralgia and myalgia and and bleeding. | 0/69 (0%) | 23/588 (3.9%) | 23/657 (3.5%) | 0.2 |
| Arthralgia and lymphadenopathy and and diarrhea. | 3/86 (3.5%) | 0/510 (0%) | 3/596 (0.5%) | 0.003 |
| Arthralgia and lymphadenopathy and nausea | 5/86 (5.8%) | 1/294 (0.3%) | 6/380 (1.6%) | 0.003 |
| Arthralgia and lymphadenopathy and vomiting | 2/86 (2.3%) | 0/517 (0%) | 2/603 (0.3%) | 0.02 |
| Arthralgia and lymphadenopathy and and bleeding. | 1/77 (1.3%) | 0/568 (0%) | 1/645 (0.2%) | 0.12 |
| Arthralgia and diarrhea and and nausea. | 4/87 (4.6%) | 1/510 (0.2%) | 5/597 (0.8%) | 0.002 |
| Arthralgia and diarrhea and and vomiting. | 4/87 (4.6%) | 36/594 (6.1%) | 40/681 (5.9%) | 0.6 |
| Arthralgia and diarrhea and Retro-orbital pain | 0/81 (0%) | 63/593 (11%) | 63/674 (9.3%) | 0.002 |
| Arthralgia and diarrhea and and bleeding. | 0/81 (0%) | 8/591 (1.4%) | 8/672 (1.2%) | 0.6 |
| Arthralgia and nausea and vomiting | 4/87 (4.6%) | 3/519 (0.6%) | 7/606 (1.2%) | 0.01 |
| Arthralgia and nausea and Retro-orbital pain | 1/76 (1.3%) | 2/405 (0.5%) | 3/481 (0.6%) | 0.4 |
| Arthralgia and vomiting and Retro-orbital pain | 0/83 (0%) | 60/595 (10%) | 60/678 (8.8%) | 0.002 |
| Arthralgia and vomiting and and bleeding. | 0/83 (0%) | 10/593 (1.7%) | 10/676 (1.5%) | 0.6 |
| Arthralgia and Retro-orbital pain bleeding | 0/61 (0%) | 20/589 (3.4%) | 20/650 (3.1%) | 0.2 |
| Edema and headache and and myalgia | 7/116 (6.0%) | 56/634 (8.8%) | 63/750 (8.4%) | 0.3 |
| Edema and headache and and lymphadenopathy | 5/117 (4.3%) | 1/578 (0.2%) | 6/695 (0.9%) | <0.001 |
| Edema and headache and and diarrhea | 5/118 (4.2%) | 21/633 (3.3%) | 26/751 (3.5%) | 0.6 |
| Edema and headache and and nausea | 7/117 (6.0%) | 3/578 (0.5%) | 10/695 (1.4%) | <0.001 |
| Edema and headache and and vomiting | 4/118 (3.4%) | 16/635 (2.5%) | 20/753 (2.7%) | 0.5 |
| Edema and headache andretro-orbital pain | 1/108 (0.9%) | 40/631 (6.3%) | 41/739 (5.5%) | 0.023 |
| Edema and headache and and bleeding | 0/106 (0%) | 8/630 (1.3%) | 8/736 (1.1%) | 0.6 |
| Edema and myalgia and lymphadenopathy | 5/117 (4.3%) | 1/573 (0.2%) | 6/690 (0.9%) | <0.001 |
| Edema and myalgia and and diarrhea | 5/118 (4.2%) | 22/634 (3.5%) | 27/752 (3.6%) | 0.6 |
| Edema and myalgia and and nausea | 6/117 (5.1%) | 3/573 (0.5%) | 9/690 (1.3%) | 0.001 |
| Edema and myalgia and and vomiting | 4/118 (3.4%) | 17/635 (2.7%) | 21/753 (2.8%) | 0.6 |
| Edema and myalgia and Retro-orbital pain | 0/107 (0%) | 42/631 (6.7%) | 42/738 (5.7%) | 0.006 |
| Edema and myalgia and and bleeding | 0/106 (0%) | 9/630 (1.4%) | 9/736 (1.2%) | 0.4 |
| Edema and lymphadenopathy and and diarrhea | 3/117 (2.6%) | 0/612 (0%) | 3/729 (0.4%) | 0.004 |
| Edema and lymphadenopathy and nausea | 4/117 (3.4%) | 1/567 (0.2%) | 5/684 (0.7%) | 0.004 |
| Edema and lymphadenopathy and vomiting | 2/117 (1.7%) | 0/618 (0%) | 2/735 (0.3%) | 0.025 |
| Edema and diarrhea and and nausea. | 4/118 (3.4%) | 1/612 (0.2%) | 5/730 (0.7%) | 0.003 |
| Edema and diarrhea and and vomiting | 4/118 (3.4%) | 6/634 (0.9%) | 10/752 (1.3%) | 0.056 |
| Edema and diarrhea and retro-orbital pain. | 0/113 (0%) | 18/632 (2.8%) | 18/745 (2.4%) | 0.092 |
| Edema and diarrhea and and bleeding | 0/112 (0%) | 3/632 (0.5%) | 3/744 (0.4%) | >0.9 |
| Edema and nausea and and vomiting | 4/118 (3.4%) | 2/619 (0.3%) | 6/737 (0.8%) | 0.007 |
| Edema and nausea and Retro-orbital pain | 1/111 (0.9%) | 1/586 (0.2%) | 2/697 (0.3%) | 0.3 |
| Edema and vomiting and Retro-orbital pain | 0/115 (0%) | 11/634 (1.7%) | 11/749 (1.5%) | 0.2 |
| Edema and vomiting and and bleeding | 0/114 (0%) | 2/633 (0.3%) | 2/747 (0.3%) | >0.9 |
| Edema and Retro-orbital pain joint dysfunction and and bleeding. | 0/101 (0%) | 8/629 (1.3%) | 8/730 (1.1%) | 0.6 |
| Headache and myalgia and and lymphadenopathy | 4/31 (13%) | 1/92 (1.1%) | 5/123 (4.1%) | 0.014 |
| Headache and Myalgia and and diarrhea | 4/32 (13%) | 112/504 (22%) | 116/536 (22%) | 0.2 |
| Headache and Myalgia and and nausea | 9/33 (27%) | 5/94 (5.3%) | 14/127 (11%) | 0.002 |
| Headache and Myalgia and and vomiting | 5/33 (15%) | 111/506 (22%) | 116/539 (22%) | 0.4 |
| Headache and myalgia andretro-orbital pain | 1/18 (5.6%) | 196/502 (39%) | 197/520 (38%) | 0.004 |
| Headache and Myalgia and and bleeding | 0/15 (0%) | 21/498 (4.2%) | 21/513 (4.1%) | >0.9 |
| Headache and lymphadenopathy and and diarrhea | 3/31 (9.7%) | 0/381 (0%) | 3/412 (0.7%) | <0.001 |
| Headache and lymphadenopathy and and nausea | 4/31 (13%) | 1/33 (3.0%) | 5/64 (7.8%) | 0.2 |
| Headache and lymphadenopathy and and vomiting | 2/31 (6.5%) | 0/388 (0%) | 2/419 (0.5%) | 0.005 |
| Headache and lymphadenopathy and and bleeding | 1/24 (4.2%) | 0/477 (0%) | 1/501 (0.2%) | 0.048 |
| Headache and diarrhea and and nausea | 5/32 (16%) | 1/381 (0.3%) | 6/413 (1.5%) | <0.001 |
| Headache and diarrhea and and vomiting | 5/32 (16%) | 59/504 (12%) | 64/536 (12%) | 0.6 |
| Headache and diarrhea andretro-orbital pain | 0/27 (0%) | 65/502 (13%) | 65/529 (12%) | 0.063 |
| Headache and diarrhea and and bleeding | 0/26 (0%) | 6/499 (1.2%) | 6/525 (1.1%) | >0.9 |
| Headache and nausea and and vomiting | 6/33 (18%) | 3/390 (0.8%) | 9/423 (2.1%) | <0.001 |
| Headache and nausea and Retro-orbital pain | 1/22 (4.5%) | 3/293 (1.0%) | 4/315 (1.3%) | 0.3 |
| Headache and vomiting andretro-orbital pain pain | 0/28 (0%) | 61/505 (12%) | 61/533 (11%) | 0.061 |
| Headache and vomiting and and bleeding | 0/27 (0%) | 9/503 (1.8%) | 9/530 (1.7%) | >0.9 |
| Headache andretro-orbital pain bleeding | 0/11 (0%) | 17/499 (3.4%) | 17/510 (3.3%) | >0.9 |
| Myalgia and lymphadenopathy and and diarrhea. | 2/31 (6.5%) | 0/389 (0%) | 2/420 (0.5%) | 0.005 |
| Myalgia and lymphadenopathy and nausea | 3/31 (9.7%) | 1/73 (1.4%) | 4/104 (3.8%) | 0.078 |
| Myalgia and lymphadenopathy and vomiting | 2/31 (6.5%) | 0/391 (0%) | 2/422 (0.5%) | 0.005 |
| Myalgia and diarrhea and and nausea. | 4/32 (13%) | 1/389 (0.3%) | 5/421 (1.2%) | <0.001 |
| Myalgia and diarrhea and and vomiting. | 4/32 (13%) | 58/504 (12%) | 62/536 (12%) | 0.8 |
| Myalgia and diarrhea and Retro-orbital pain | 0/28 (0%) | 64/503 (13%) | 64/531 (12%) | 0.037 |
| Myalgia and diarrhea and and bleeding | 0/27 (0%) | 8/501 (1.6%) | 8/528 (1.5%) | >0.9 |
| Myalgia and nausea and vomiting | 5/33 (15%) | 3/393 (0.8%) | 8/426 (1.9%) | <0.001 |
| Myalgia and nausea and Retro-orbital pain | 0/23 (0%) | 2/301 (0.7%) | 2/324 (0.6%) | >0.9 |
| Myalgia and vomiting and Retro-orbital pain | 0/29 (0%) | 64/505 (13%) | 64/534 (12%) | 0.037 |
| Myalgia and vomiting and and bleeding. | 0/28 (0%) | 10/503 (2.0%) | 10/531 (1.9%) | >0.9 |
| Myalgia and Retro-orbital pain and bleeding | 0/12 (0%) | 20/499 (4.0%) | 20/511 (3.9%) | >0.9 |
| Lymphadenopathy and diarrhea and and nausea. | 2/31 (6.5%) | 0/376 (0%) | 2/407 (0.5%) | 0.006 |
| Lymphadenopathy and diarrhea and and vomiting. | 2/31 (6.5%) | 0/444 (0%) | 2/475 (0.4%) | 0.004 |
| Lymphadenopathy and nausea and vomiting | 2/31 (6.5%) | 0/382 (0%) | 2/413 (0.5%) | 0.005 |
| Diarrhea and nausea and and vomiting | 5/32 (16%) | 1/444 (0.2%) | 6/476 (1.3%) | <0.001 |
| Diarrhea and vomiting and retro-orbital pain | 0/28 (0%) | 28/503 (5.6%) | 28/531 (5.3%) | 0.4 |
| Diarrhea and vomiting and and bleeding | 0/27 (0%) | 3/503 (0.6%) | 3/530 (0.6%) | >0.9 |
| Diarrhea and Retro-orbital pain bleeding | 0/26 (0%) | 7/502 (1.4%) | 7/528 (1.3%) | >0.9 |
| Nausea and vomiting and Retro-orbital pain | 0/28 (0%) | 2/442 (0.5%) | 2/470 (0.4%) | >0.9 |
| Vomiting and Retro-orbital pain and bleeding | 0/28 (0%) | 9/503 (1.8%) | 9/531 (1.7%) | >0.9 |

^1^ n (%)

2

**Supplementary Table 3. Sensitivity, specificity, and predictive values of clinical signs and symptoms for RT-qPCR-confirmed ZIKV infection.**

| Signs and symptoms | TP^1^ | FP^2^ | FN^3^ | TN^4^ | Sensitivity % (95% CI)^5^ | Specificity % (95% CI)^5^ | PPV^6^ %  (95% CI)^5^ | NPV % (IC95%) | Acurácia % (IC95%) |
| --- | --- | --- | --- | --- | --- | --- | --- | --- | --- |
| Fever | 265 | 749 | 53 | 53 | 83.3 (78.8–87.3) | 6.6 (5–8.6) | 26.1 (23.5–29) | 50 (40.1–59.9) | 28.4 (25.8–31.1) |
| Pruritus | 300 | 365 | 19 | 437 | 94 (90.9–96.4) | 54.5 (51–58) | 45.1 (41.3–49) | 95.8 (93.6–97.5) | 65.7 (62.9–68.5) |
| Conjunctivitis | 231 | 212 | 86 | 588 | 72.9 (67.6–77.7) | 73.5 (70.3–76.5) | 52.1 (47.4–56.9) | 87.2 (84.5–89.7) | 73.3 (70.6–75.9) |
| Arthralgia | 258 | 513 | 61 | 289 | 80.9 (76.1–85) | 36 (32.7–39.5) | 33.5 (30.1–36.9) | 82.6 (78.2–86.4) | 48.8 (45.8–51.8) |
| Edema | 216 | 235 | 100 | 563 | 68.4 (62.9–73.4) | 70.6 (67.3–73.7) | 47.9 (43.2–52.6) | 84.9 (82–87.6) | 69.9 (67.1–72.6) |
| Headache | 24 | 476 | 9 | 29 | 72.7 (54.5–86.7) | 5.7 (3.9–8.1) | 4.8 (3.1–7.1) | 76.3 (59.8–88.6) | 9.9 (7.5–12.8) |
| Myalgia | 23 | 438 | 10 | 68 | 69.7 (51.3–84.4) | 13.4 (10.6–16.7) | 5 (3.2–7.4) | 87.2 (77.7–93.7) | 16.9 (13.9–20.4) |
| Lymphadenopathy | 10 | 1 | 21 | 5 | 32.3 (16.7–51.4) | 83.3 (35.9–99.6) | 90.9 (58.7–99.8) | 19.2 (6.6–39.4) | 40.5 (25.2–57.8) |
| Diarrhea | 7 | 128 | 25 | 375 | 21.9 (9.3–40) | 74.6 (70.5–78.3) | 5.2 (2.1–10.4) | 93.8 (90.9–95.9) | 71.4 (67.3–75.2) |
| Nausea | 15 | 6 | 18 | 3 | 45.5 (28.1–63.6) | 33.3 (7.5–70.1) | 71.4 (47.8–88.7) | 14.3 (3–36.3) | 42.9 (28.1–58.9) |
| Vomiting | 6 | 125 | 27 | 381 | 18.2 (7–35.5) | 75.3 (71.3–79) | 4.6 (1.7–9.7) | 93.4 (90.5–95.6) | 71.8 (67.8–75.5) |
| Retro-orbital pain | 3 | 224 | 2 | 276 | 60 (14.7–94.7) | 55.2 (50.7–59.6) | 1.3 (0.3–3.8) | 99.3 (97.4–99.9) | 55.2 (50.8–59.6) |
| Fever and Pruritus | 252 | 327 | 66 | 475 | 79.2 (74.4–83.6) | 59.2 (55.7–62.7) | 43.5 (39.4–47.7) | 87.8 (84.7–90.4) | 64.9 (62–67.7) |
| Fever and conjunctivitis | 194 | 196 | 123 | 604 | 61.2 (55.6–66.6) | 75.5 (72.4–78.4) | 49.7 (44.7–54.8) | 83.1 (80.2–85.7) | 71.4 (68.7–74.1) |
| Fever and arthralgia | 216 | 481 | 102 | 321 | 67.9 (62.5–73) | 40 (36.6–43.5) | 31 (27.6–34.6) | 75.9 (71.5–79.9) | 47.9 (45–50.9) |
| Fever and edema | 182 | 212 | 133 | 586 | 57.8 (52.1–63.3) | 73.4 (70.2–76.5) | 46.2 (41.2–51.3) | 81.5 (78.5–84.3) | 69 (66.2–71.7) |
| Fever and headache | 20 | 468 | 59 | 78 | 25.3 (16.2–36.4) | 14.3 (11.5–17.5) | 4.1 (2.5–6.3) | 56.9 (48.2–65.4) | 15.7 (13–18.8) |
| Fever and myalgia | 17 | 429 | 61 | 118 | 21.8 (13.2–32.6) | 21.6 (18.2–25.3) | 3.8 (2.2–6) | 65.9 (58.5–72.8) | 21.6 (18.5–25.1) |
| Fever and lymphadenopathy | 10 | 1 | 67 | 57 | 13 (6.4–22.6) | 98.3 (90.8–100) | 90.9 (58.7–99.8) | 46 (37–55.1) | 49.6 (41–58.3) |
| Fever and diarrhea | 6 | 125 | 71 | 419 | 7.8 (2.9–16.2) | 77 (73.3–80.5) | 4.6 (1.7–9.7) | 85.5 (82.1–88.5) | 68.4 (64.6–72) |
| Fever and nausea | 13 | 6 | 66 | 55 | 16.5 (9.1–26.5) | 90.2 (79.8–96.3) | 68.4 (43.4–87.4) | 45.5 (36.4–54.8) | 48.6 (40.1–57.1) |
| Fever and vomiting | 6 | 123 | 73 | 424 | 7.6 (2.8–15.8) | 77.5 (73.8–80.9) | 4.7 (1.7–9.8) | 85.3 (81.9–88.3) | 68.7 (64.9–72.3) |
| Fever and Retro-orbital pain | 2 | 220 | 54 | 322 | 3.6 (0.4–12.3) | 59.4 (55.1–63.6) | 0.9 (0.1–3.2) | 85.6 (81.7–89) | 54.2 (50.1–58.2) |
| Fever and bleeding | 1 | 25 | 53 | 511 | 1.9 (0–9.9) | 95.3 (93.2–97) | 3.8 (0.1–19.6) | 90.6 (87.9–92.9) | 86.8 (83.7–89.4) |
| Pruritus and conjunctivitis | 217 | 164 | 100 | 638 | 68.5 (63–73.5) | 79.6 (76.6–82.3) | 57 (51.8–62) | 86.4 (83.8–88.8) | 76.4 (73.8–78.8) |
| Pruritus and arthralgia | 246 | 272 | 73 | 530 | 77.1 (72.1–81.6) | 66.1 (62.7–69.4) | 47.5 (43.1–51.9) | 87.9 (85–90.4) | 69.2 (66.4–71.9) |
| Pruritus and edema | 206 | 189 | 110 | 611 | 65.2 (59.7–70.4) | 76.4 (73.3–79.3) | 52.2 (47.1–57.2) | 84.7 (81.9–87.3) | 73.2 (70.5–75.8) |
| Pruritus and headache | 22 | 112 | 27 | 452 | 44.9 (30.7–59.8) | 80.1 (76.6–83.4) | 16.4 (10.6–23.8) | 94.4 (91.9–96.3) | 77.3 (73.8–80.5) |
| Pruritus and myalgia | 22 | 115 | 27 | 449 | 44.9 (30.7–59.8) | 79.6 (76–82.9) | 16.1 (10.3–23.3) | 94.3 (91.9–96.2) | 76.8 (73.2–80.1) |
| Pruritus and lymphadenopathy | 10 | 1 | 37 | 440 | 21.3 (10.7–35.7) | 99.8 (98.7–100) | 90.9 (58.7–99.8) | 92.2 (89.5–94.5) | 92.2 (89.4–94.4) |
| Pruritus and diarrhea | 6 | 41 | 42 | 522 | 12.5 (4.7–25.2) | 92.7 (90.3–94.7) | 12.8 (4.8–25.7) | 92.6 (90.1–94.6) | 86.4 (83.4–89) |
| Pruritus and nausea | 14 | 3 | 35 | 439 | 28.6 (16.6–43.3) | 99.3 (98–99.9) | 82.4 (56.6–96.2) | 92.6 (89.9–94.8) | 92.3 (89.4–94.4) |
| Pruritus and vomiting | 5 | 36 | 44 | 528 | 10.2 (3.4–22.2) | 93.6 (91.3–95.5) | 12.2 (4.1–26.2) | 92.3 (89.8–94.4) | 86.9 (84–89.5) |
| Pruritus and Retro-orbital pain | 1 | 86 | 21 | 474 | 4.5 (0.1–22.8) | 84.6 (81.4–87.5) | 1.1 (0–6.2) | 95.8 (93.6–97.4) | 81.6 (78.2–84.6) |
| Pruritus and bleeding | 1 | 17 | 19 | 542 | 5 (0.1–24.9) | 97 (95.2–98.2) | 5.6 (0.1–27.3) | 96.6 (94.8–97.9) | 93.8 (91.4–95.5) |
| Conjunctivitis and arthralgia | 192 | 177 | 125 | 624 | 60.6 (55–66) | 77.9 (74.9–80.7) | 52 (46.8–57.2) | 83.3 (80.4–85.9) | 73 (70.3–75.6) |
| Conjunctivitis and edema | 169 | 120 | 147 | 679 | 53.5 (47.8–59.1) | 85 (82.3–87.4) | 58.5 (52.6–64.2) | 82.2 (79.4–84.8) | 76.1 (73.4–78.5) |
| Conjunctivitis and headache | 15 | 62 | 92 | 596 | 14 (8.1–22.1) | 90.6 (88.1–92.7) | 19.5 (11.3–30.1) | 86.6 (83.9–89.1) | 79.9 (76.8–82.6) |
| Conjunctivitis and myalgia | 13 | 61 | 94 | 596 | 12.1 (6.6–19.9) | 90.7 (88.2–92.8) | 17.6 (9.7–28.2) | 86.4 (83.6–88.8) | 79.7 (76.6–82.5) |
| Conjunctivitis and lymphadenopathy | 10 | 1 | 98 | 591 | 9.3 (4.5–16.4) | 99.8 (99.1–100) | 90.9 (58.7–99.8) | 85.8 (82.9–88.3) | 85.9 (83–88.3) |
| Conjunctivitis and diarrhea | 4 | 25 | 104 | 632 | 3.7 (1–9.2) | 96.2 (94.4–97.5) | 13.8 (3.9–31.7) | 85.9 (83.1–88.3) | 83.1 (80.3–85.7) |
| Conjunctivitis and nausea | 10 | 4 | 98 | 589 | 9.3 (4.5–16.4) | 99.3 (98.3–99.8) | 71.4 (41.9–91.6) | 85.7 (82.9–88.3) | 85.4 (82.6–87.9) |
| Conjunctivitis and vomiting | 4 | 16 | 105 | 643 | 3.7 (1–9.1) | 97.6 (96.1–98.6) | 20 (5.7–43.7) | 86 (83.3–88.4) | 84.2 (81.4–86.7) |
| Conjunctivitis and Retro-orbital pain | 2 | 52 | 88 | 602 | 2.2 (0.3–7.8) | 92 (89.7–94) | 3.7 (0.5–12.7) | 87.2 (84.5–89.6) | 81.2 (78.1–83.9) |
| Conjunctivitis and bleeding | 1 | 6 | 86 | 648 | 1.1 (0–6.2) | 99.1 (98–99.7) | 14.3 (0.4–57.9) | 88.3 (85.7–90.5) | 87.6 (84.9–89.8) |
| Arthralgia and edema | 192 | 194 | 125 | 606 | 60.6 (55–66) | 75.8 (72.6–78.7) | 49.7 (44.6–54.8) | 82.9 (80–85.6) | 71.4 (68.7–74.1) |
| Arthralgia and headache | 18 | 291 | 69 | 305 | 20.7 (12.7–30.7) | 51.2 (47.1–55.3) | 5.8 (3.5–9.1) | 81.6 (77.2–85.4) | 47.3 (43.5–51.1) |
| Arthralgia and myalgia | 18 | 284 | 69 | 312 | 20.7 (12.7–30.7) | 52.3 (48.3–56.4) | 6 (3.6–9.3) | 81.9 (77.6–85.6) | 48.3 (44.5–52.1) |
| Arthralgia and lymphadenopathy | 10 | 1 | 76 | 293 | 11.6 (5.7–20.3) | 99.7 (98.1–100) | 90.9 (58.7–99.8) | 79.4 (74.9–83.4) | 79.7 (75.3–83.6) |
| Arthralgia and diarrhea | 6 | 85 | 81 | 509 | 6.9 (2.6–14.4) | 85.7 (82.6–88.4) | 6.6 (2.5–13.8) | 86.3 (83.2–88.9) | 75.6 (72.2–78.8) |
| Arthralgia and nausea | 12 | 5 | 75 | 291 | 13.8 (7.3–22.9) | 98.3 (96.1–99.4) | 70.6 (44–89.7) | 79.5 (75–83.5) | 79.1 (74.6–83) |
| Arthralgia and vomiting | 4 | 80 | 83 | 516 | 4.6 (1.3–11.4) | 86.6 (83.6–89.2) | 4.8 (1.3–11.7) | 86.1 (83.1–88.8) | 76.1 (72.7–79.2) |
| Arthralgia and Retro-orbital pain | 3 | 190 | 61 | 401 | 4.7 (1–13.1) | 67.9 (63.9–71.6) | 1.6 (0.3–4.5) | 86.8 (83.4–89.7) | 61.7 (57.8–65.4) |
| Arthralgia and bleeding | 1 | 23 | 61 | 564 | 1.6 (0–8.7) | 96.1 (94.2–97.5) | 4.2 (0.1–21.1) | 90.2 (87.6–92.5) | 87.1 (84.2–89.5) |
| Edema and headache | 10 | 58 | 106 | 575 | 8.6 (4.2–15.3) | 90.8 (88.3–93) | 14.7 (7.3–25.4) | 84.4 (81.5–87.1) | 78.1 (74.9–81) |
| Edema and myalgia | 10 | 64 | 106 | 569 | 8.6 (4.2–15.3) | 89.9 (87.3–92.1) | 13.5 (6.7–23.5) | 84.3 (81.3–87) | 77.3 (74.1–80.2) |
| Edema and lymphadenopathy | 7 | 1 | 110 | 566 | 6 (2.4–11.9) | 99.8 (99–100) | 87.5 (47.3–99.7) | 83.7 (80.7–86.4) | 83.8 (80.7–86.4) |
| Edema and diarrhea | 6 | 22 | 112 | 611 | 5.1 (1.9–10.7) | 96.5 (94.8–97.8) | 21.4 (8.3–41) | 84.5 (81.7–87.1) | 82.2 (79.2–84.8) |
| Edema and nausea | 8 | 3 | 109 | 564 | 6.8 (3–13) | 99.5 (98.5–99.9) | 72.7 (39–94) | 83.8 (80.8–86.5) | 83.6 (80.6–86.3) |
| Edema and vomiting | 4 | 18 | 114 | 617 | 3.4 (0.9–8.5) | 97.2 (95.6–98.3) | 18.2 (5.2–40.3) | 84.4 (81.6–87) | 82.5 (79.5–85.1) |
| Edema and Retro-orbital pain | 2 | 46 | 101 | 584 | 1.9 (0.2–6.8) | 92.7 (90.4–94.6) | 4.2 (0.5–14.3) | 85.3 (82.4–87.8) | 79.9 (76.8–82.7) |
| Headache and myalgia | 18 | 417 | 15 | 88 | 54.5 (36.4–71.9) | 17.4 (14.2–21) | 4.1 (2.5–6.5) | 85.4 (77.1–91.6) | 19.7 (16.5–23.4) |
| Headache and lymphadenopathy | 8 | 1 | 23 | 32 | 25.8 (11.9–44.6) | 97 (84.2–99.9) | 88.9 (51.8–99.7) | 58.2 (44.1–71.3) | 62.5 (49.5–74) |
| Headache and diarrhea | 6 | 123 | 26 | 380 | 18.8 (7.2–36.4) | 75.5 (71.5–79.2) | 4.7 (1.7–9.8) | 93.6 (90.8–95.8) | 72.1 (68.1–75.9) |
| Headache and nausea | 13 | 6 | 20 | 30 | 39.4 (22.9–57.9) | 83.3 (67.2–93.6) | 68.4 (43.4–87.4) | 60 (45.2–73.6) | 62.3 (49.8–73.5) |
| Headache and vomiting | 6 | 119 | 27 | 387 | 18.2 (7–35.5) | 76.5 (72.5–80.1) | 4.8 (1.8–10.2) | 93.5 (90.7–95.7) | 72.9 (68.9–76.6) |
| Headache andretro-orbital pain | 2 | 213 | 11 | 289 | 15.4 (1.9–45.4) | 57.6 (53.1–61.9) | 0.9 (0.1–3.3) | 96.3 (93.5–98.2) | 56.5 (52.1–60.8) |
| Headache and bleeding | 1 | 22 | 9 | 474 | 10 (0.3–44.5) | 95.6 (93.4–97.2) | 4.3 (0.1–21.9) | 98.1 (96.5–99.1) | 93.9 (91.3–95.7) |
| Myalgia and lymphadenopathy | 6 | 1 | 25 | 72 | 19.4 (7.5–37.5) | 98.6 (92.6–100) | 85.7 (42.1–99.6) | 74.2 (64.3–82.6) | 75 (65.4–82.7) |
| Myalgia and diarrhea | 5 | 116 | 27 | 388 | 15.6 (5.3–32.8) | 77 (73.1–80.6) | 4.1 (1.4–9.4) | 93.5 (90.7–95.7) | 73.3 (69.3–77) |
| Myalgia and nausea | 11 | 5 | 22 | 70 | 33.3 (18–51.8) | 93.3 (85.1–97.8) | 68.8 (41.3–89) | 76.1 (66.1–84.4) | 75 (65.6–82.6) |
| Myalgia and vomiting | 5 | 116 | 28 | 390 | 15.2 (5.1–31.9) | 77.1 (73.2–80.7) | 4.1 (1.4–9.4) | 93.3 (90.5–95.5) | 73.3 (69.3–76.9) |
| Myalgia and Retro-orbital pain | 1 | 204 | 12 | 297 | 7.7 (0.2–36) | 59.3 (54.8–63.6) | 0.5 (0–2.7) | 96.1 (93.3–98) | 58 (53.6–62.3) |
| Lymphadenopathy and nausea | 5 | 1 | 26 | 5 | 16.1 (5.5–33.7) | 83.3 (35.9–99.6) | 83.3 (35.9–99.6) | 16.1 (5.5–33.7) | 27 (14.4–44.4) |
| Diarrhea and nausea | 5 | 1 | 27 | 375 | 15.6 (5.3–32.8) | 99.7 (98.5–100) | 83.3 (35.9–99.6) | 93.3 (90.4–95.5) | 93.1 (90.1–95.3) |
| Diarrhea and vomiting | 5 | 61 | 27 | 443 | 15.6 (5.3–32.8) | 87.9 (84.7–90.6) | 7.6 (2.5–16.8) | 94.3 (91.8–96.2) | 83.6 (80.1–86.6) |
| Nausea and vomiting | 6 | 3 | 27 | 381 | 18.2 (7–35.5) | 99.2 (97.7–99.8) | 66.7 (29.9–92.5) | 93.4 (90.5–95.6) | 92.8 (89.8–95) |
| Nausea and Retro-orbital pain | 1 | 3 | 19 | 279 | 5 (0.1–24.9) | 98.9 (96.9–99.8) | 25 (0.6–80.6) | 93.6 (90.2–96.1) | 92.7 (89–95.3) |
| Fever and Pruritus and conjunctivitis | 185 | 151 | 132 | 651 | 58.4 (52.7–63.8) | 81.2 (78.3–83.8) | 55.1 (49.6–60.5) | 83.1 (80.3–85.7) | 74.7 (72–77.2) |
| Fever and Pruritus and Arthralgia | 209 | 251 | 109 | 551 | 65.7 (60.2–70.9) | 68.7 (65.4–71.9) | 45.4 (40.8–50.1) | 83.5 (80.4–86.2) | 67.9 (65–70.6) |
| Fever and Pruritus and edema | 176 | 171 | 139 | 629 | 55.9 (50.2–61.4) | 78.6 (75.6–81.4) | 50.7 (45.3–56.1) | 81.9 (79–84.6) | 72.2 (69.4–74.8) |
| Fever and Pruritus and headache | 18 | 108 | 72 | 486 | 20 (12.3–29.8) | 81.8 (78.5–84.8) | 14.3 (8.7–21.6) | 87.1 (84–89.8) | 73.7 (70.2–76.9) |
| Fever and Pruritus and Myalgia | 16 | 110 | 73 | 484 | 18 (10.6–27.5) | 81.5 (78.1–84.5) | 12.7 (7.4–19.8) | 86.9 (83.8–89.6) | 73.2 (69.7–76.5) |
| Fever and Pruritus and lymphadenopathy | 10 | 1 | 78 | 477 | 11.4 (5.6–19.9) | 99.8 (98.8–100) | 90.9 (58.7–99.8) | 85.9 (82.8–88.7) | 86 (82.8–88.7) |
| Fever and Pruritus and diarrhea | 5 | 40 | 83 | 553 | 5.7 (1.9–12.8) | 93.3 (90.9–95.1) | 11.1 (3.7–24.1) | 86.9 (84.1–89.5) | 81.9 (78.8–84.7) |
| Fever and Pruritus and nausea | 12 | 3 | 78 | 476 | 13.3 (7.1–22.1) | 99.4 (98.2–99.9) | 80 (51.9–95.7) | 85.9 (82.7–88.7) | 85.8 (82.6–88.5) |
| Fever and Pruritus and vomiting | 5 | 36 | 85 | 558 | 5.6 (1.8–12.5) | 93.9 (91.7–95.7) | 12.2 (4.1–26.2) | 86.8 (83.9–89.3) | 82.3 (79.2–85.1) |
| Fever and pruritus and Retro-orbital pain | 1 | 84 | 67 | 507 | 1.5 (0–7.9) | 85.8 (82.7–88.5) | 1.2 (0–6.4) | 88.3 (85.4–90.8) | 77.1 (73.6–80.2) |
| Fever and Pruritus and bleeding | 1 | 17 | 66 | 573 | 1.5 (0–8) | 97.1 (95.4–98.3) | 5.6 (0.1–27.3) | 89.7 (87–91.9) | 87.4 (84.5–89.8) |
| Fever and conjunctivitis and arthralgia | 164 | 163 | 153 | 638 | 51.7 (46.1–57.4) | 79.7 (76.7–82.4) | 50.2 (44.6–55.7) | 80.7 (77.7–83.4) | 71.7 (69–74.3) |
| Fever and conjunctivitis and edema | 145 | 112 | 171 | 687 | 45.9 (40.3–51.6) | 86 (83.4–88.3) | 56.4 (50.1–62.6) | 80.1 (77.2–82.7) | 74.6 (71.9–77.1) |
| Fever and conjunctivitis and headache | 13 | 61 | 126 | 609 | 9.4 (5.1–15.5) | 90.9 (88.5–93) | 17.6 (9.7–28.2) | 82.9 (79.9–85.5) | 76.9 (73.8–79.7) |
| Fever and conjunctivitis and myalgia | 10 | 59 | 129 | 610 | 7.2 (3.5–12.8) | 91.2 (88.8–93.2) | 14.5 (7.2–25) | 82.5 (79.6–85.2) | 76.7 (73.6–79.6) |
| Fever and conjunctivitis and lymphadenopathy | 10 | 1 | 130 | 607 | 7.1 (3.5–12.7) | 99.8 (99.1–100) | 90.9 (58.7–99.8) | 82.4 (79.4–85) | 82.5 (79.5–85.1) |
| Fever and conjunctivitis and diarrhea | 4 | 25 | 136 | 644 | 2.9 (0.8–7.2) | 96.3 (94.5–97.6) | 13.8 (3.9–31.7) | 82.6 (79.7–85.2) | 80.1 (77.1–82.8) |
| Fever and conjunctivitis and nausea | 9 | 4 | 131 | 605 | 6.4 (3–11.9) | 99.3 (98.3–99.8) | 69.2 (38.6–90.9) | 82.2 (79.2–84.9) | 82 (79–84.6) |
| Fever and conjunctivitis and vomiting | 4 | 16 | 137 | 655 | 2.8 (0.8–7.1) | 97.6 (96.2–98.6) | 20 (5.7–43.7) | 82.7 (79.9–85.3) | 81.2 (78.3–83.8) |
| Fever and conjunctivitis and Retro-orbital pain | 1 | 50 | 124 | 616 | 0.8 (0–4.4) | 92.5 (90.2–94.4) | 2 (0–10.4) | 83.2 (80.4–85.9) | 78 (74.9–80.8) |
| Fever and conjunctivitis and bleeding | 1 | 6 | 123 | 660 | 0.8 (0–4.4) | 99.1 (98–99.7) | 14.3 (0.4–57.9) | 84.3 (81.6–86.8) | 83.7 (80.9–86.1) |
| Fever and arthralgia and edema | 162 | 176 | 154 | 624 | 51.3 (45.6–56.9) | 78 (75–80.8) | 47.9 (42.5–53.4) | 80.2 (77.2–83) | 70.4 (67.6–73.1) |
| Fever and Arthralgia and headache | 16 | 285 | 107 | 333 | 13 (7.6–20.3) | 53.9 (49.9–57.9) | 5.3 (3.1–8.5) | 75.7 (71.4–79.6) | 47.1 (43.5–50.8) |
| Fever and arthralgia and myalgia | 14 | 277 | 108 | 341 | 11.5 (6.4–18.5) | 55.2 (51.2–59.1) | 4.8 (2.7–7.9) | 75.9 (71.7–79.8) | 48 (44.3–51.6) |
| Fever and arthralgia and lymphadenopathy | 10 | 1 | 112 | 324 | 8.2 (4–14.6) | 99.7 (98.3–100) | 90.9 (58.7–99.8) | 74.3 (69.9–78.4) | 74.7 (70.4–78.6) |
| Fever and Arthralgia and diarrhea | 5 | 83 | 117 | 533 | 4.1 (1.3–9.3) | 86.5 (83.6–89.1) | 5.7 (1.9–12.8) | 82 (78.8–84.9) | 72.9 (69.5–76) |
| Fever and arthralgia and nausea | 10 | 5 | 113 | 322 | 8.1 (4–14.4) | 98.5 (96.5–99.5) | 66.7 (38.4–88.2) | 74 (69.6–78.1) | 73.8 (69.4–77.7) |
| Fever and arthralgia and vomiting | 4 | 79 | 119 | 539 | 3.3 (0.9–8.1) | 87.2 (84.3–89.7) | 4.8 (1.3–11.9) | 81.9 (78.8–84.8) | 73.3 (69.9–76.4) |
| Fever and arthralgia and Retro-orbital pain | 2 | 186 | 102 | 428 | 1.9 (0.2–6.8) | 69.7 (65.9–73.3) | 1.1 (0.1–3.8) | 80.8 (77.1–84) | 59.9 (56.2–63.5) |
| Fever and arthralgia and bleeding | 1 | 23 | 102 | 587 | 1 (0–5.3) | 96.2 (94.4–97.6) | 4.2 (0.1–21.1) | 85.2 (82.3–87.8) | 82.5 (79.4–85.1) |
| Fever and edema and headache | 9 | 54 | 137 | 595 | 6.2 (2.9–11.4) | 91.7 (89.3–93.7) | 14.3 (6.7–25.4) | 81.3 (78.3–84) | 76 (72.8–78.9) |
| Fever and edema and myalgia | 8 | 59 | 137 | 590 | 5.5 (2.4–10.6) | 90.9 (88.4–93) | 11.9 (5.3–22.2) | 81.2 (78.1–83.9) | 75.3 (72.1–78.2) |
| Fever and edema and lymphadenopathy | 7 | 1 | 140 | 589 | 4.8 (1.9–9.6) | 99.8 (99.1–100) | 87.5 (47.3–99.7) | 80.8 (77.7–83.6) | 80.9 (77.8–83.6) |
| Fever and edema and diarrhea | 5 | 21 | 142 | 628 | 3.4 (1.1–7.8) | 96.8 (95.1–98) | 19.2 (6.6–39.4) | 81.6 (78.6–84.2) | 79.5 (76.5–82.2) |
| Fever and edema and nausea | 8 | 3 | 139 | 587 | 5.4 (2.4–10.4) | 99.5 (98.5–99.9) | 72.7 (39–94) | 80.9 (77.8–83.7) | 80.7 (77.7–83.5) |
| Fever and edema and vomiting | 4 | 18 | 144 | 633 | 2.7 (0.7–6.8) | 97.2 (95.7–98.4) | 18.2 (5.2–40.3) | 81.5 (78.6–84.1) | 79.7 (76.7–82.4) |
| Fever and edema and Retro-orbital pain | 1 | 44 | 134 | 602 | 0.7 (0–4.1) | 93.2 (91–95) | 2.2 (0.1–11.8) | 81.8 (78.8–84.5) | 77.2 (74.1–80.1) |
| Fever and headache and Myalgia | 14 | 409 | 65 | 137 | 17.7 (10–27.9) | 25.1 (21.5–28.9) | 3.3 (1.8–5.5) | 67.8 (60.9–74.2) | 24.2 (20.9–27.8) |
| Fever and headache and lymphadenopathy | 8 | 1 | 69 | 81 | 10.4 (4.6–19.4) | 98.8 (93.4–100) | 88.9 (51.8–99.7) | 54 (45.7–62.2) | 56 (47.9–63.8) |
| Fever and headache and diarrhea | 6 | 120 | 72 | 424 | 7.7 (2.9–16) | 77.9 (74.2–81.4) | 4.8 (1.8–10.1) | 85.5 (82.1–88.5) | 69.1 (65.3–72.7) |
| Fever and headache and nausea | 12 | 6 | 67 | 79 | 15.2 (8.1–25) | 92.9 (85.3–97.4) | 66.7 (41–86.7) | 54.1 (45.7–62.4) | 55.5 (47.5–63.2) |
| Fever and headache and vomiting | 6 | 117 | 73 | 430 | 7.6 (2.8–15.8) | 78.6 (74.9–82) | 4.9 (1.8–10.3) | 85.5 (82.1–88.4) | 69.6 (65.9–73.2) |
| Fever and headache andretro-orbital pain pain | 2 | 211 | 60 | 332 | 3.2 (0.4–11.2) | 61.1 (56.9–65.3) | 0.9 (0.1–3.4) | 84.7 (80.7–88.1) | 55.2 (51.1–59.2) |
| Fever and headache and bleeding | 1 | 22 | 59 | 515 | 1.7 (0–8.9) | 95.9 (93.9–97.4) | 4.3 (0.1–21.9) | 89.7 (86.9–92.1) | 86.4 (83.4–89) |
| Fever and myalgia and lymphadenopathy | 6 | 1 | 71 | 122 | 7.8 (2.9–16.2) | 99.2 (95.6–100) | 85.7 (42.1–99.6) | 63.2 (56–70) | 64 (56.9–70.6) |
| Fever and Myalgia and diarrhea | 4 | 113 | 73 | 432 | 5.2 (1.4–12.8) | 79.3 (75.6–82.6) | 3.4 (0.9–8.5) | 85.5 (82.2–88.5) | 70.1 (66.3–73.6) |
| Fever and Myalgia and nausea | 9 | 5 | 70 | 120 | 11.4 (5.3–20.5) | 96 (90.9–98.7) | 64.3 (35.1–87.2) | 63.2 (55.9–70) | 63.2 (56.2–69.8) |
| Fever and Myalgia and vomiting | 5 | 114 | 74 | 433 | 6.3 (2.1–14.2) | 79.2 (75.5–82.5) | 4.2 (1.4–9.5) | 85.4 (82–88.4) | 70 (66.2–73.5) |
| Fever and myalgia and Retro-orbital pain | 1 | 202 | 62 | 340 | 1.6 (0–8.5) | 62.7 (58.5–66.8) | 0.5 (0–2.7) | 84.6 (80.7–88) | 56.4 (52.3–60.3) |
| Fever and lymphadenopathy and nausea | 5 | 1 | 72 | 57 | 6.5 (2.1–14.5) | 98.3 (90.8–100) | 83.3 (35.9–99.6) | 44.2 (35.5–53.2) | 45.9 (37.4–54.7) |
| Fever and diarrhea and nausea | 5 | 1 | 73 | 419 | 6.4 (2.1–14.3) | 99.8 (98.7–100) | 83.3 (35.9–99.6) | 85.2 (81.7–88.2) | 85.1 (81.6–88.1) |
| Fever and diarrhea and vomiting | 5 | 59 | 73 | 486 | 6.4 (2.1–14.3) | 89.2 (86.3–91.7) | 7.8 (2.6–17.3) | 86.9 (83.9–89.6) | 78.8 (75.3–81.9) |
| Fever and nausea and vomiting | 6 | 3 | 73 | 424 | 7.6 (2.8–15.8) | 99.3 (98–99.9) | 66.7 (29.9–92.5) | 85.3 (81.9–88.3) | 85 (81.5–87.9) |
| Fever and nausea and Retro-orbital pain | 1 | 3 | 67 | 324 | 1.5 (0–7.9) | 99.1 (97.3–99.8) | 25 (0.6–80.6) | 82.9 (78.8–86.5) | 82.3 (78.1–85.8) |
| Pruritus and conjunctivitis and arthralgia | 183 | 137 | 134 | 665 | 57.7 (52.1–63.2) | 82.9 (80.1–85.5) | 57.2 (51.6–62.7) | 83.2 (80.5–85.8) | 75.8 (73.1–78.2) |
| Pruritus and conjunctivitis and edema | 159 | 103 | 157 | 697 | 50.3 (44.7–56) | 87.1 (84.6–89.4) | 60.7 (54.5–66.6) | 81.6 (78.9–84.2) | 76.7 (74.1–79.1) |
| Pruritus and conjunctivitis and headache | 15 | 39 | 105 | 645 | 12.5 (7.2–19.8) | 94.3 (92.3–95.9) | 27.8 (16.5–41.6) | 86 (83.3–88.4) | 82.1 (79.2–84.6) |
| Pruritus and conjunctivitis and myalgia | 13 | 41 | 107 | 643 | 10.8 (5.9–17.8) | 94 (92–95.7) | 24.1 (13.5–37.6) | 85.7 (83–88.2) | 81.6 (78.7–84.2) |
| Pruritus and conjunctivitis and lymphadenopathy | 10 | 1 | 111 | 639 | 8.3 (4–14.7) | 99.8 (99.1–100) | 90.9 (58.7–99.8) | 85.2 (82.5–87.7) | 85.3 (82.5–87.7) |
| Pruritus and conjunctivitis and diarrhea | 4 | 15 | 117 | 668 | 3.3 (0.9–8.2) | 97.8 (96.4–98.8) | 21.1 (6.1–45.6) | 85.1 (82.4–87.5) | 83.6 (80.8–86) |
| Pruritus and conjunctivitis and nausea | 10 | 3 | 111 | 638 | 8.3 (4–14.7) | 99.5 (98.6–99.9) | 76.9 (46.2–95) | 85.2 (82.4–87.6) | 85 (82.3–87.5) |
| Pruritus and conjunctivitis and vomiting | 4 | 9 | 118 | 675 | 3.3 (0.9–8.2) | 98.7 (97.5–99.4) | 30.8 (9.1–61.4) | 85.1 (82.4–87.5) | 84.2 (81.5–86.7) |
| Pruritus and conjunctivitis andretro-orbital pain | 1 | 34 | 102 | 648 | 1 (0–5.3) | 95 (93.1–96.5) | 2.9 (0.1–14.9) | 86.4 (83.7–88.8) | 82.7 (79.8–85.2) |
| Pruritus and conjunctivitis and bleeding | 1 | 6 | 100 | 675 | 1 (0–5.4) | 99.1 (98.1–99.7) | 14.3 (0.4–57.9) | 87.1 (84.5–89.4) | 86.4 (83.8–88.7) |
| Pruritus and Arthralgia and edema | 184 | 158 | 133 | 643 | 58 (52.4–63.5) | 80.3 (77.3–83) | 53.8 (48.4–59.2) | 82.9 (80–85.4) | 74 (71.3–76.5) |
| Pruritus and Arthralgia and headache | 17 | 93 | 80 | 541 | 17.5 (10.6–26.6) | 85.3 (82.3–88) | 15.5 (9.3–23.6) | 87.1 (84.2–89.7) | 76.3 (73–79.3) |
| Pruritus and arthralgia and myalgia | 17 | 98 | 80 | 536 | 17.5 (10.6–26.6) | 84.5 (81.5–87.3) | 14.8 (8.9–22.6) | 87 (84.1–89.6) | 75.6 (72.3–78.7) |
| Pruritus and arthralgia and lymphadenopathy | 10 | 1 | 86 | 533 | 10.4 (5.1–18.3) | 99.8 (99–100) | 90.9 (58.7–99.8) | 86.1 (83.1–88.7) | 86.2 (83.2–88.7) |
| Pruritus and Arthralgia and diarrhea | 6 | 38 | 91 | 596 | 6.2 (2.3–13) | 94 (91.9–95.7) | 13.6 (5.2–27.4) | 86.8 (84–89.2) | 82.4 (79.4–85) |
| Pruritus and arthralgia and nausea | 12 | 2 | 85 | 532 | 12.4 (6.6–20.6) | 99.6 (98.7–100) | 85.7 (57.2–98.2) | 86.2 (83.2–88.8) | 86.2 (83.2–88.8) |
| Pruritus and Arthralgia and vomiting | 4 | 29 | 93 | 605 | 4.1 (1.1–10.2) | 95.4 (93.5–96.9) | 12.1 (3.4–28.2) | 86.7 (83.9–89.1) | 83.3 (80.4–85.9) |
| Pruritus and arthralgia andretro-orbital pain pain | 1 | 79 | 73 | 551 | 1.4 (0–7.3) | 87.5 (84.6–89.9) | 1.2 (0–6.8) | 88.3 (85.5–90.7) | 78.4 (75.1–81.4) |
| Pruritus and Arthralgia and bleeding | 1 | 17 | 73 | 613 | 1.4 (0–7.3) | 97.3 (95.7–98.4) | 5.6 (0.1–27.3) | 89.4 (86.8–91.6) | 87.2 (84.5–89.5) |
| Pruritus and Edema and headache | 10 | 42 | 115 | 620 | 8 (3.9–14.2) | 93.7 (91.5–95.4) | 19.2 (9.6–32.5) | 84.4 (81.5–86.9) | 80.1 (77–82.8) |
| Pruritus and edema and myalgia | 10 | 47 | 115 | 616 | 8 (3.9–14.2) | 92.9 (90.7–94.7) | 17.5 (8.7–29.9) | 84.3 (81.4–86.8) | 79.4 (76.4–82.2) |
| Pruritus and edema and lymphadenopathy | 7 | 1 | 119 | 612 | 5.6 (2.3–11.1) | 99.8 (99.1–100) | 87.5 (47.3–99.7) | 83.7 (80.8–86.3) | 83.8 (80.9–86.3) |
| Pruritus and Edema and diarrhea | 6 | 18 | 121 | 645 | 4.7 (1.8–10) | 97.3 (95.7–98.4) | 25 (9.8–46.7) | 84.2 (81.4–86.7) | 82.4 (79.5–85) |
| Pruritus and Edema and nausea | 8 | 1 | 118 | 611 | 6.3 (2.8–12.1) | 99.8 (99.1–100) | 88.9 (51.8–99.7) | 83.8 (80.9–86.4) | 83.9 (81–86.4) |
| Pruritus and Edema and vomiting | 4 | 14 | 123 | 650 | 3.1 (0.9–7.9) | 97.9 (96.5–98.8) | 22.2 (6.4–47.6) | 84.1 (81.3–86.6) | 82.7 (79.8–85.2) |
| Pruritus and edema and Retro-orbital pain | 1 | 36 | 111 | 625 | 0.9 (0–4.9) | 94.6 (92.5–96.2) | 2.7 (0.1–14.2) | 84.9 (82.1–87.4) | 81 (78–83.7) |
| Pruritus and headache and Myalgia | 17 | 106 | 32 | 458 | 34.7 (21.7–49.6) | 81.2 (77.7–84.3) | 13.8 (8.3–21.2) | 93.5 (90.9–95.5) | 77.5 (73.9–80.7) |
| Pruritus and headache and lymphadenopathy | 8 | 1 | 39 | 454 | 17 (7.6–30.8) | 99.8 (98.8–100) | 88.9 (51.8–99.7) | 92.1 (89.3–94.3) | 92 (89.2–94.2) |
| Pruritus and headache and diarrhea | 5 | 38 | 43 | 525 | 10.4 (3.5–22.7) | 93.3 (90.9–95.2) | 11.6 (3.9–25.1) | 92.4 (89.9–94.5) | 86.7 (83.7–89.3) |
| Pruritus and headache and nausea | 12 | 3 | 37 | 453 | 24.5 (13.3–38.9) | 99.3 (98.1–99.9) | 80 (51.9–95.7) | 92.4 (89.7–94.6) | 92.1 (89.3–94.2) |
| Pruritus and headache and vomiting | 5 | 31 | 44 | 533 | 10.2 (3.4–22.2) | 94.5 (92.3–96.2) | 13.9 (4.7–29.5) | 92.4 (89.9–94.4) | 87.8 (84.8–90.2) |
| Pruritus and headache andretro-orbital pain pain | 1 | 76 | 29 | 485 | 3.3 (0.1–17.2) | 86.5 (83.3–89.2) | 1.3 (0–7) | 94.4 (92–96.2) | 82.2 (78.9–85.2) |
| Pruritus and headache and bleeding | 1 | 14 | 27 | 546 | 3.6 (0.1–18.3) | 97.5 (95.8–98.6) | 6.7 (0.2–31.9) | 95.3 (93.2–96.9) | 93 (90.6–94.9) |
| Pruritus and myalgia and lymphadenopathy | 6 | 1 | 41 | 451 | 12.8 (4.8–25.7) | 99.8 (98.8–100) | 85.7 (42.1–99.6) | 91.7 (88.9–94) | 91.6 (88.7–93.8) |
| Pruritus and Myalgia and diarrhea | 5 | 39 | 43 | 525 | 10.4 (3.5–22.7) | 93.1 (90.7–95) | 11.4 (3.8–24.6) | 92.4 (89.9–94.5) | 86.6 (83.6–89.1) |
| Pruritus and myalgia and nausea | 11 | 2 | 38 | 450 | 22.4 (11.8–36.6) | 99.6 (98.4–99.9) | 84.6 (54.6–98.1) | 92.2 (89.5–94.4) | 92 (89.2–94.2) |
| Pruritus and Myalgia and vomiting | 5 | 35 | 44 | 529 | 10.2 (3.4–22.2) | 93.8 (91.5–95.6) | 12.5 (4.2–26.8) | 92.3 (89.8–94.4) | 87.1 (84.1–89.6) |
| Pruritus and lymphadenopathy and nausea | 5 | 1 | 42 | 440 | 10.6 (3.5–23.1) | 99.8 (98.7–100) | 83.3 (35.9–99.6) | 91.3 (88.4–93.6) | 91.2 (88.2–93.5) |
| Pruritus and diarrhea and vomiting | 4 | 17 | 44 | 547 | 8.3 (2.3–20) | 97 (95.2–98.2) | 19 (5.4–41.9) | 92.6 (90.1–94.5) | 90 (87.3–92.2) |
| Pruritus and nausea and Retro-orbital pain | 1 | 1 | 36 | 476 | 2.7 (0.1–14.2) | 99.8 (98.8–100) | 50 (1.3–98.7) | 93 (90.4–95) | 92.8 (90.1–94.8) |
| Conjunctivitis and arthralgia and edema | 154 | 110 | 163 | 691 | 48.6 (43–54.2) | 86.3 (83.7–88.6) | 58.3 (52.1–64.3) | 80.9 (78.1–83.5) | 75.6 (72.9–78.1) |
| Conjunctivitis and arthralgia and headache | 11 | 51 | 130 | 630 | 7.8 (4–13.5) | 92.5 (90.3–94.4) | 17.7 (9.2–29.5) | 82.9 (80–85.5) | 78 (75–80.7) |
| Conjunctivitis and arthralgia and myalgia | 9 | 51 | 132 | 630 | 6.4 (3–11.8) | 92.5 (90.3–94.4) | 15 (7.1–26.6) | 82.7 (79.8–85.3) | 77.7 (74.7–80.5) |
| Conjunctivitis and arthralgia and lymphadenopathy | 10 | 1 | 133 | 626 | 7 (3.4–12.5) | 99.8 (99.1–100) | 90.9 (58.7–99.8) | 82.5 (79.6–85.1) | 82.6 (79.7–85.2) |
| Conjunctivitis and arthralgia and diarrhea | 4 | 20 | 139 | 661 | 2.8 (0.8–7) | 97.1 (95.5–98.2) | 16.7 (4.7–37.4) | 82.6 (79.8–85.2) | 80.7 (77.8–83.3) |
| Conjunctivitis and arthralgia and nausea | 9 | 3 | 133 | 624 | 6.3 (2.9–11.7) | 99.5 (98.6–99.9) | 75 (42.8–94.5) | 82.4 (79.5–85.1) | 82.3 (79.4–84.9) |
| Conjunctivitis and arthralgia and vomiting | 3 | 16 | 140 | 666 | 2.1 (0.4–6) | 97.7 (96.2–98.7) | 15.8 (3.4–39.6) | 82.6 (79.8–85.2) | 81.1 (78.2–83.7) |
| Conjunctivitis and arthralgia and pain and retroorbital | 2 | 44 | 125 | 634 | 1.6 (0.2–5.6) | 93.5 (91.4–95.2) | 4.3 (0.5–14.8) | 83.5 (80.7–86.1) | 79 (76–81.7) |
| Conjunctivitis and arthralgia and bleeding | 1 | 6 | 125 | 673 | 0.8 (0–4.3) | 99.1 (98.1–99.7) | 14.3 (0.4–57.9) | 84.3 (81.6–86.8) | 83.7 (81–86.2) |
| Conjunctivitis and edema and headache | 8 | 26 | 151 | 685 | 5 (2.2–9.7) | 96.3 (94.7–97.6) | 23.5 (10.7–41.2) | 81.9 (79.2–84.5) | 79.7 (76.8–82.3) |
| Conjunctivitis and edema and myalgia | 7 | 27 | 152 | 684 | 4.4 (1.8–8.9) | 96.2 (94.5–97.5) | 20.6 (8.7–37.9) | 81.8 (79–84.4) | 79.4 (76.6–82) |
| Conjunctivitis and edema and lymphadenopathy | 7 | 1 | 154 | 682 | 4.3 (1.8–8.8) | 99.9 (99.2–100) | 87.5 (47.3–99.7) | 81.6 (78.8–84.2) | 81.6 (78.8–84.2) |
| Conjunctivitis and edema and diarrhea | 4 | 12 | 157 | 700 | 2.5 (0.7–6.2) | 98.3 (97.1–99.1) | 25 (7.3–52.4) | 81.7 (78.9–84.2) | 80.6 (77.8–83.2) |
| Conjunctivitis and edema and nausea | 7 | 2 | 153 | 680 | 4.4 (1.8–8.8) | 99.7 (98.9–100) | 77.8 (40–97.2) | 81.6 (78.8–84.2) | 81.6 (78.8–84.1) |
| Conjunctivitis and edema and vomiting | 3 | 7 | 158 | 706 | 1.9 (0.4–5.3) | 99 (98–99.6) | 30 (6.7–65.2) | 81.7 (79–84.2) | 81.1 (78.3–83.6) |
| Conjunctivitis and edema and Retro-orbital pain | 2 | 23 | 148 | 685 | 1.3 (0.2–4.7) | 96.8 (95.2–97.9) | 8 (1–26) | 82.2 (79.5–84.8) | 80.1 (77.2–82.7) |
| Conjunctivitis and headache and myalgia | 10 | 56 | 97 | 602 | 9.3 (4.6–16.5) | 91.5 (89.1–93.5) | 15.2 (7.5–26.1) | 86.1 (83.3–88.6) | 80 (77–82.7) |
| Conjunctivitis and headache and lymphadenopathy | 8 | 1 | 100 | 598 | 7.4 (3.3–14.1) | 99.8 (99.1–100) | 88.9 (51.8–99.7) | 85.7 (82.9–88.2) | 85.7 (82.9–88.2) |
| Conjunctivitis and headache and diarrhea | 4 | 24 | 104 | 633 | 3.7 (1–9.2) | 96.3 (94.6–97.6) | 14.3 (4–32.7) | 85.9 (83.2–88.3) | 83.3 (80.4–85.8) |
| Conjunctivitis and headache and nausea | 8 | 4 | 100 | 596 | 7.4 (3.3–14.1) | 99.3 (98.3–99.8) | 66.7 (34.9–90.1) | 85.6 (82.8–88.2) | 85.3 (82.4–87.8) |
| Conjunctivitis and headache and vomiting | 4 | 14 | 105 | 645 | 3.7 (1–9.1) | 97.9 (96.5–98.8) | 22.2 (6.4–47.6) | 86 (83.3–88.4) | 84.5 (81.7–87) |
| Conjunctivitis and headache andretro-orbital pain | 1 | 47 | 94 | 608 | 1.1 (0–5.7) | 92.8 (90.6–94.7) | 2.1 (0.1–11.1) | 86.6 (83.9–89) | 81.2 (78.2–83.9) |
| Conjunctivitis and headache and bleeding | 1 | 4 | 92 | 651 | 1.1 (0–5.8) | 99.4 (98.4–99.8) | 20 (0.5–71.6) | 87.6 (85–89.9) | 87.2 (84.5–89.4) |
| Conjunctivitis and myalgia and lymphadenopathy | 6 | 1 | 102 | 599 | 5.6 (2.1–11.7) | 99.8 (99.1–100) | 85.7 (42.1–99.6) | 85.4 (82.6–88) | 85.5 (82.6–87.9) |
| Conjunctivitis and myalgia and diarrhea | 3 | 23 | 105 | 635 | 2.8 (0.6–7.9) | 96.5 (94.8–97.8) | 11.5 (2.4–30.2) | 85.8 (83.1–88.2) | 83.3 (80.4–85.8) |
| Conjunctivitis and myalgia and nausea | 7 | 3 | 101 | 597 | 6.5 (2.6–12.9) | 99.5 (98.5–99.9) | 70 (34.8–93.3) | 85.5 (82.7–88.1) | 85.3 (82.4–87.8) |
| Conjunctivitis and myalgia and vomiting | 4 | 16 | 105 | 643 | 3.7 (1–9.1) | 97.6 (96.1–98.6) | 20 (5.7–43.7) | 86 (83.3–88.4) | 84.2 (81.4–86.7) |
| Conjunctivitis and lymphadenopathy and nausea | 5 | 1 | 103 | 591 | 4.6 (1.5–10.5) | 99.8 (99.1–100) | 83.3 (35.9–99.6) | 85.2 (82.3–87.7) | 85.1 (82.2–87.7) |
| Conjunctivitis and diarrhea and nausea | 3 | 1 | 105 | 632 | 2.8 (0.6–7.9) | 99.8 (99.1–100) | 75 (19.4–99.4) | 85.8 (83–88.2) | 85.7 (82.9–88.1) |
| Conjunctivitis and diarrhea and vomiting | 3 | 7 | 105 | 652 | 2.8 (0.6–7.9) | 98.9 (97.8–99.6) | 30 (6.7–65.2) | 86.1 (83.5–88.5) | 85.4 (82.7–87.8) |
| Conjunctivitis and nausea and vomiting | 4 | 1 | 105 | 643 | 3.7 (1–9.1) | 99.8 (99.1–100) | 80 (28.4–99.5) | 86 (83.3–88.4) | 85.9 (83.2–88.3) |
| Conjunctivitis and nausea and Retro-orbital pain | 1 | 1 | 99 | 603 | 1 (0–5.4) | 99.8 (99.1–100) | 50 (1.3–98.7) | 85.9 (83.1–88.4) | 85.8 (82.9–88.2) |
| Arthralgia and edema and headache. | 10 | 53 | 131 | 615 | 7.1 (3.5–12.7) | 92.1 (89.8–94) | 15.9 (7.9–27.3) | 82.4 (79.5–85.1) | 77.3 (74.2–80.1) |
| Arthralgia and edema and myalgia | 10 | 58 | 131 | 610 | 7.1 (3.5–12.7) | 91.3 (88.9–93.3) | 14.7 (7.3–25.4) | 82.3 (79.4–85) | 76.6 (73.5–79.5) |
| Arthralgia and edema and lymphadenopathy | 7 | 1 | 135 | 608 | 4.9 (2–9.9) | 99.8 (99.1–100) | 87.5 (47.3–99.7) | 81.8 (78.9–84.5) | 81.9 (78.9–84.5) |
| Arthralgia and edema and diarrhea | 6 | 22 | 137 | 646 | 4.2 (1.6–8.9) | 96.7 (95.1–97.9) | 21.4 (8.3–41) | 82.5 (79.7–85.1) | 80.4 (77.5–83) |
| Arthralgia and edema and nausea | 8 | 3 | 134 | 606 | 5.6 (2.5–10.8) | 99.5 (98.6–99.9) | 72.7 (39–94) | 81.9 (78.9–84.6) | 81.8 (78.8–84.4) |
| Arthralgia and edema and vomiting | 4 | 14 | 139 | 655 | 2.8 (0.8–7) | 97.9 (96.5–98.9) | 22.2 (6.4–47.6) | 82.5 (79.7–85.1) | 81.2 (78.3–83.8) |
| Arthralgia and edema and Retro-orbital pain | 2 | 43 | 125 | 623 | 1.6 (0.2–5.6) | 93.5 (91.4–95.3) | 4.4 (0.5–15.1) | 83.3 (80.4–85.9) | 78.8 (75.8–81.6) |
| Arthralgia and headache and myalgia | 13 | 271 | 74 | 325 | 14.9 (8.2–24.2) | 54.5 (50.4–58.6) | 4.6 (2.5–7.7) | 81.5 (77.3–85.1) | 49.5 (45.7–53.3) |
| Arthralgia and headache and lymphadenopathy | 8 | 1 | 78 | 308 | 9.3 (4.1–17.5) | 99.7 (98.2–100) | 88.9 (51.8–99.7) | 79.8 (75.4–83.7) | 80 (75.6–83.8) |
| Arthralgia and headache and diarrhea. | 5 | 82 | 82 | 512 | 5.7 (1.9–12.9) | 86.2 (83.2–88.9) | 5.7 (1.9–12.9) | 86.2 (83.2–88.9) | 75.9 (72.5–79) |
| Arthralgia and headache and nausea | 10 | 5 | 77 | 306 | 11.5 (5.7–20.1) | 98.4 (96.3–99.5) | 66.7 (38.4–88.2) | 79.9 (75.5–83.8) | 79.4 (75–83.2) |
| Arthralgia and headache and vomiting | 4 | 76 | 83 | 520 | 4.6 (1.3–11.4) | 87.2 (84.3–89.8) | 5 (1.4–12.3) | 86.2 (83.2–88.9) | 76.7 (73.3–79.8) |
| Arthralgia and headache andretro-orbital pain | 2 | 180 | 69 | 412 | 2.8 (0.3–9.8) | 69.6 (65.7–73.3) | 1.1 (0.1–3.9) | 85.7 (82.2–88.7) | 62.4 (58.6–66.1) |
| Arthralgia and headache and bleeding. | 1 | 20 | 69 | 568 | 1.4 (0–7.7) | 96.6 (94.8–97.9) | 4.8 (0.1–23.8) | 89.2 (86.5–91.5) | 86.5 (83.6–88.9) |
| Arthralgia and myalgia and lymphadenopathy | 6 | 1 | 80 | 315 | 7 (2.6–14.6) | 99.7 (98.2–100) | 85.7 (42.1–99.6) | 79.7 (75.4–83.6) | 79.9 (75.5–83.6) |
| Arthralgia and myalgia and diarrhea | 5 | 82 | 82 | 512 | 5.7 (1.9–12.9) | 86.2 (83.2–88.9) | 5.7 (1.9–12.9) | 86.2 (83.2–88.9) | 75.9 (72.5–79) |
| Arthralgia and myalgia and nausea | 9 | 5 | 78 | 313 | 10.3 (4.8–18.7) | 98.4 (96.4–99.5) | 64.3 (35.1–87.2) | 80.1 (75.7–83.9) | 79.5 (75.2–83.3) |
| Arthralgia and myalgia and vomiting | 4 | 78 | 83 | 518 | 4.6 (1.3–11.4) | 86.9 (83.9–89.5) | 4.9 (1.3–12) | 86.2 (83.2–88.8) | 76.4 (73–79.5) |
| Arthralgia and myalgia and Retro-orbital pain | 1 | 179 | 69 | 413 | 1.4 (0–7.7) | 69.8 (65.9–73.4) | 0.6 (0–3.1) | 85.7 (82.2–88.7) | 62.5 (58.7–66.2) |
| Arthralgia and lymphadenopathy and nausea | 5 | 1 | 81 | 293 | 5.8 (1.9–13) | 99.7 (98.1–100) | 83.3 (35.9–99.6) | 78.3 (73.8–82.4) | 78.4 (73.9–82.4) |
| Arthralgia and diarrhea and nausea. | 4 | 1 | 83 | 509 | 4.6 (1.3–11.4) | 99.8 (98.9–100) | 80 (28.4–99.5) | 86 (82.9–88.7) | 85.9 (82.8–88.6) |
| Arthralgia and diarrhea and vomiting. | 4 | 36 | 83 | 558 | 4.6 (1.3–11.4) | 93.9 (91.7–95.7) | 10 (2.8–23.7) | 87.1 (84.2–89.6) | 82.5 (79.4–85.3) |
| Arthralgia and nausea and vomiting | 4 | 3 | 83 | 516 | 4.6 (1.3–11.4) | 99.4 (98.3–99.9) | 57.1 (18.4–90.1) | 86.1 (83.1–88.8) | 85.8 (82.7–88.4) |
| Arthralgia and nausea and Retro-orbital pain | 1 | 2 | 75 | 403 | 1.3 (0–7.1) | 99.5 (98.2–99.9) | 33.3 (0.8–90.6) | 84.3 (80.7–87.5) | 84 (80.3–87.1) |
| Edema and headache and myalgia | 7 | 56 | 109 | 578 | 6 (2.5–12) | 91.2 (88.7–93.3) | 11.1 (4.6–21.6) | 84.1 (81.2–86.8) | 78 (74.8–80.9) |
| Edema and headache and lymphadenopathy | 5 | 1 | 112 | 577 | 4.3 (1.4–9.7) | 99.8 (99–100) | 83.3 (35.9–99.6) | 83.7 (80.8–86.4) | 83.7 (80.7–86.4) |
| Edema and headache and diarrhea | 5 | 21 | 113 | 612 | 4.2 (1.4–9.6) | 96.7 (95–97.9) | 19.2 (6.6–39.4) | 84.4 (81.6–87) | 82.2 (79.2–84.8) |
| Edema and headache and nausea | 7 | 3 | 110 | 575 | 6 (2.4–11.9) | 99.5 (98.5–99.9) | 70 (34.8–93.3) | 83.9 (81–86.6) | 83.7 (80.7–86.4) |
| Edema and headache and vomiting | 4 | 16 | 114 | 619 | 3.4 (0.9–8.5) | 97.5 (95.9–98.6) | 20 (5.7–43.7) | 84.4 (81.6–87) | 82.7 (79.8–85.3) |
| Edema and headache andretro-orbital pain | 1 | 40 | 107 | 591 | 0.9 (0–5.1) | 93.7 (91.5–95.4) | 2.4 (0.1–12.9) | 84.7 (81.8–87.3) | 80.1 (77–82.9) |
| Edema and myalgia and lymphadenopathy | 5 | 1 | 112 | 572 | 4.3 (1.4–9.7) | 99.8 (99–100) | 83.3 (35.9–99.6) | 83.6 (80.6–86.3) | 83.6 (80.6–86.3) |
| Edema and myalgia and diarrhea | 5 | 22 | 113 | 612 | 4.2 (1.4–9.6) | 96.5 (94.8–97.8) | 18.5 (6.3–38.1) | 84.4 (81.6–87) | 82 (79.1–84.7) |
| Edema and myalgia and nausea | 6 | 3 | 111 | 570 | 5.1 (1.9–10.8) | 99.5 (98.5–99.9) | 66.7 (29.9–92.5) | 83.7 (80.7–86.4) | 83.5 (80.4–86.1) |
| Edema and myalgia and vomiting | 4 | 17 | 114 | 618 | 3.4 (0.9–8.5) | 97.3 (95.7–98.4) | 19 (5.4–41.9) | 84.4 (81.6–87) | 82.6 (79.7–85.2) |
| Edema and lymphadenopathy and nausea | 4 | 1 | 113 | 566 | 3.4 (0.9–8.5) | 99.8 (99–100) | 80 (28.4–99.5) | 83.4 (80.3–86.1) | 83.3 (80.3–86) |
| Edema and diarrhea and nausea. | 4 | 1 | 114 | 611 | 3.4 (0.9–8.5) | 99.8 (99.1–100) | 80 (28.4–99.5) | 84.3 (81.4–86.9) | 84.2 (81.4–86.8) |
| Edema and diarrhea and vomiting | 4 | 6 | 114 | 628 | 3.4 (0.9–8.5) | 99.1 (98–99.7) | 40 (12.2–73.8) | 84.6 (81.8–87.2) | 84 (81.2–86.5) |
| Edema and nausea and vomiting | 4 | 2 | 114 | 617 | 3.4 (0.9–8.5) | 99.7 (98.8–100) | 66.7 (22.3–95.7) | 84.4 (81.6–87) | 84.3 (81.4–86.8) |
| Edema and nausea and Retro-orbital pain | 1 | 1 | 110 | 585 | 0.9 (0–4.9) | 99.8 (99.1–100) | 50 (1.3–98.7) | 84.2 (81.2–86.8) | 84.1 (81.1–86.7) |
| Headache and myalgia and lymphadenopathy | 4 | 1 | 27 | 91 | 12.9 (3.6–29.8) | 98.9 (94.1–100) | 80 (28.4–99.5) | 77.1 (68.5–84.3) | 77.2 (68.6–84.1) |
| Headache and Myalgia and diarrhea | 4 | 112 | 28 | 392 | 12.5 (3.5–29) | 77.8 (73.9–81.3) | 3.4 (0.9–8.6) | 93.3 (90.5–95.5) | 73.9 (69.9–77.5) |
| Headache and Myalgia and nausea | 9 | 5 | 24 | 89 | 27.3 (13.3–45.5) | 94.7 (88–98.3) | 64.3 (35.1–87.2) | 78.8 (70.1–85.9) | 77.2 (68.7–83.9) |
| Headache and Myalgia and vomiting | 5 | 111 | 28 | 395 | 15.2 (5.1–31.9) | 78.1 (74.2–81.6) | 4.3 (1.4–9.8) | 93.4 (90.6–95.6) | 74.2 (70.3–77.8) |
| Headache and myalgia andretro-orbital pain | 1 | 196 | 17 | 306 | 5.6 (0.1–27.3) | 61 (56.5–65.2) | 0.5 (0–2.8) | 94.7 (91.7–96.9) | 59 (54.7–63.3) |
| Headache and lymphadenopathy and nausea | 4 | 1 | 27 | 32 | 12.9 (3.6–29.8) | 97 (84.2–99.9) | 80 (28.4–99.5) | 54.2 (40.8–67.3) | 56.2 (43.3–68.4) |
| Headache and diarrhea and nausea | 5 | 1 | 27 | 380 | 15.6 (5.3–32.8) | 99.7 (98.5–100) | 83.3 (35.9–99.6) | 93.4 (90.5–95.6) | 93.2 (90.2–95.4) |
| Headache and diarrhea and vomiting | 5 | 59 | 27 | 445 | 15.6 (5.3–32.8) | 88.3 (85.2–91) | 7.8 (2.6–17.3) | 94.3 (91.8–96.2) | 84 (80.5–86.9) |
| Headache and nausea and vomiting | 6 | 3 | 27 | 387 | 18.2 (7–35.5) | 99.2 (97.8–99.8) | 66.7 (29.9–92.5) | 93.5 (90.7–95.7) | 92.9 (89.9–95.1) |
| Headache and nausea and Retro-orbital pain | 1 | 3 | 21 | 290 | 4.5 (0.1–22.8) | 99 (97–99.8) | 25 (0.6–80.6) | 93.2 (89.9–95.8) | 92.4 (88.7–95) |
| Myalgia and lymphadenopathy and nausea | 3 | 1 | 28 | 72 | 9.7 (2–25.8) | 98.6 (92.6–100) | 75 (19.4–99.4) | 72 (62.1–80.5) | 72.1 (62.3–80.2) |
| Myalgia and diarrhea and nausea. | 4 | 1 | 28 | 388 | 12.5 (3.5–29) | 99.7 (98.6–100) | 80 (28.4–99.5) | 93.3 (90.4–95.5) | 93.1 (90.1–95.3) |
| Myalgia and diarrhea and vomiting | 4 | 58 | 28 | 446 | 12.5 (3.5–29) | 88.5 (85.4–91.1) | 6.5 (1.8–15.7) | 94.1 (91.6–96) | 84 (80.5–86.9) |
| Myalgia and nausea and vomiting | 5 | 3 | 28 | 390 | 15.2 (5.1–31.9) | 99.2 (97.8–99.8) | 62.5 (24.5–91.5) | 93.3 (90.5–95.5) | 92.7 (89.7–94.9) |
| Diarrhea and nausea and vomiting | 5 | 1 | 27 | 443 | 15.6 (5.3–32.8) | 99.8 (98.8–100) | 83.3 (35.9–99.6) | 94.3 (91.8–96.2) | 94.1 (91.5–96) |

^1^True Positive

^2^False Positive

^3^False Negative

^4^True Negative

^5^95% confidence interval

^6^Positive predictive value

^7^Negative predictive value.
